# Ni-Based Molecular Sieves Nanomaterials for Dry Methane Reforming: Role of Porous Structure and Active Sites Distribution on Hydrogen Production

**DOI:** 10.3390/nano14151320

**Published:** 2024-08-05

**Authors:** Ahmed S. Al-Fatesh, Ahmed A. Ibrahim, Anis H. Fakeeha, Ahmed I. Osman, Yousef M. Alanazi, Fahad Saleh Almubaddel, Ahmed E. Abasaeed

**Affiliations:** 1 Chemical Engineering Department, College of Engineering, King Saud University, P.O. Box 800, Riyadh 11421, Saudi Arabia; aalfaesh@ksu.edu.sa (A.S.A.-F.); aidid@ksu.edu.sa (A.A.I.); anishf@ksu.edu.sa (A.H.F.); yalanazi1@ksu.edu.sa (Y.M.A.); abasaeed@ksu.edu.sa (A.E.A.); 2 School of Chemistry and Chemical Engineering, Queen’s University Belfast, Belfast BT9 5AG, Northern Ireland, UK

**Keywords:** molecular sieve nanomaterial, silica–alumina ratio, dry reforming of methane, nickel active sites, respond surface methodology, central composite design

## Abstract

Global warming, driven by greenhouse gases like CH_4_ and CO_2_, necessitates efficient catalytic conversion to syngas. Herein, Ni containing different molecular sieve nanomaterials are investigated for dry reforming of methane (DRM). The reduced catalysts are characterized by surface area porosity, X-ray diffraction, Raman infrared spectroscopy, CO_2_ temperature-programmed desorption techniques, and transmission electron microscopy. The active sites over each molecular sieve remain stable under oxidizing gas CO_2_ during DRM. The reduced 5Ni/CBV10A catalyst, characterized by the lowest silica–alumina ratio, smallest surface area and pore volume, and narrow 8-ring connecting channels, generated the maximum number of active sites on its outer surface. In contrast, the reduced-5Ni/CBV3024E catalyst, with the highest silica–alumina ratio, more than double the surface area and pore volume, 12-ring sinusoidal porous channels, and smallest Ni crystallite, produced the highest H_2_ output (44%) after 300 min of operation at 700 °C, with a CH_4_:CO_2_ = 1:1, P = 1 atom, gas hour space velocity (GHSV) = 42 L gcat^−1^ h^−1^. This performance was achieved despite having 25% fewer initial active sites, suggesting that a larger fraction of these sites is stabilized within the pore channels, leading to sustained catalytic activity. Using central composite design and response surface methodology, we successfully optimized the process conditions for the 5Ni/CBV3024E catalyst. The optimized conditions yielded a desirable H_2_ to CO ratio of 1.00, with a H_2_ yield of 91.92% and a CO yield of 89.16%, indicating high efficiency in gas production. The experimental results closely aligned with the predicted values, demonstrating the effectiveness of the optimization approach.

## 1. Introduction

The climate emergency requires significant efforts for effective mitigation. Greenhouse gases (GHGs), such as carbon dioxide and methane, contribute to 90% of total emissions, exacerbating global warming [1]. Global temperature increases lead to biodiversity loss, extreme weather events and glacier melting [2,3]. Industrial activities have significantly contributed to GHGs, aggravating climate change. As a result, the research on converting CH_4_ and CO_2_ into H_2_ and CO (syngas) has garnered significant global interest. This process, known as the dry reforming of methane (DRM), produces syngas, which has significant applications in both synthetic processes and energy production [4].

**Figure 1:**
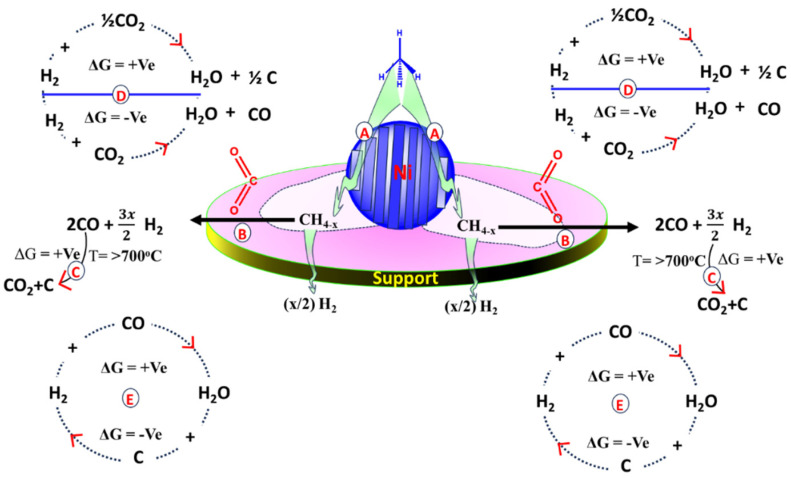
The detailed reaction scheme of DRM and other parallel reactions over supported Ni-based catalyst.

Cost-effective Ni and Co as active sites are preferred over costly noble metal active sites; whereas, in the mean of CH_4_ interaction energy, Ni is preferred over Co [5,6]. Catalytic materials at the nanoscale often have unique catalytic properties to the bulk [7], like strong adsorption energy for CH_4_ and CO_2_, easy CO_2_ dissociation, and excellent carbon elimination [7]. Maria et al. observed that upon moving from bulk to nano, the infrared vibration frequency shifted to a higher magnitude (500 cm^−1^ to 570 cm^−1^), indicating enhanced metal-support interaction at the nano level [8]. The detailed reaction scheme of DRM and other parallel reactions is shown in Figure 1. The DRM reaction can be summed up by two steps, namely dissociation of CH_4_ over edge-step sites of Ni and oxidation of dissociated methane (CH_4_-x; x = 1–4) into syngas by CO_2_ [9,10] (Figure 1A,B). The DRM reaction is highly endothermic and takes place within 550–850 °C. Within this temperature range, various parallel competitive reactions also run over the catalyst surface which can alter the final H_2_ yield and CO yield ratio over the catalyst. Carbon monoxide disproportionation reaction (Boudouard reaction; 2CO → CO_2_ +C) is a side reaction and is responsible for carbon deposit. However, by adjusting a temperature of ~700 °C, this reaction becomes thermodynamically infeasible [11] (Figure 1C). The interaction of H_2_ (one of the DRM products) with CO_2_ cannot be neglected (Figure 1D). Especially, a reverse water gas shift reaction (H_2_ +CO_2_ → H_2_O +CO) is thermodynamically feasible, which gives water and CO. The consumption of H_2_ and the formation of CO in RWGS reactions affect the H_2_/CO ratio seriously. However, the gasification of carbon by water (C + H_2_O → CO + H_2_) further improves the H_2_ and CO yield during the DRM reaction (Figure 1E).

Alumina is typical support for Ni active sites, but it has limitations for DRM due to its high acidity and the tendency of Ni to migrate into the alumina. Zeolites, which are aluminosilicates, offer a better alternative because they have a rigid microporous framework that supports Ni active sites effectively [12,13]. The unique arrangement of pores and windows in zeolites facilitates the diffusion of small molecules, including reactants and products. Ni/USY catalysts show higher reducibility, while Ni/ZSM-5 has a stronger metal-support interaction compared to Ni/Al_2_O_3_ [14,15,16,17], leading to higher activity and better stability. Synthetic aluminosilicate molecular sieves, which mimic natural zeolites, have tunable pore sizes based on their silica–alumina ratio, allowing for optimization through specific synthetic strategies [18,19].

In addition to using powerful catalysts, achieving optimal catalytic activity in DRM involves fine-tuning various operating parameters such as temperature, the CH_4_/CO_2_ ratio, and space velocity. However, finding the best combination of these parameters through experimentation is labor-intensive, time-consuming, and energy-demanding. Thankfully, statistical tools like response surface methodology (RSM) can predict the optimal catalytic performance using minimal experimental data. RSM continuously exhibits a high level of predictive power, with modeled and experimental results closely matching. RSM includes several designs, such as the Box–Behnken design (BBD), central composite design (CCD), and Daehler design (DD) [20]. For instance, RSM predicted a 30.98% H_2_ yield and 69.36% CO yield for an yttrium oxide-cobalt/mesoporous alumina catalyst at 900 °C reaction temperature, 30,690 mLgcat^−1^h^−1^ WHSV, and 1.027 CH_4_/CO_2_ [21]. Experimental results under the same conditions showed yields of 32.12% H_2_ and 67.88% CO. Similarly, for calcium iron oxide-supported nickel catalyst, RSM predicted 77.82% H_2_ yield and 75.76% CO yield at 832.45 °C reaction temperature, 0.96 CH_4_/CO_2_ ratio, and 35,000 mL g^−1^ h^−1^ gas hour space velocity [22], closely matching experimental results.

These close correlations between predictions and experiments were also observed for other catalysts, including Ta-promoted Ni/ZSM-5, alumina–magnesia supported Ni-W, and dendritic fibrous SBA-15-supported Ni catalysts, using the central composite design under RSM [23,24,25]. Today, RSM is not limited to optimizing the reaction parameters but also to fine-tuning the catalyst’s composition, such as the proportion of active sites, promoters, and supports, to achieve the best catalytic activity for DRM reactions [26].

The work aims to produce a new, practical, and dependable Ni-based catalyst for the DRM process supported by synthetic molecular sieves with a distinct pore structure and a variable silica–alumina ratio. Unlike conventional techniques, this novel approach seeks to improve catalytic performance and stability. Several sophisticated instrumentation techniques are used to confirm the catalytic activity results, such as X-ray diffraction, surface area porosity, Raman infrared spectroscopy, and temperature-programmed reduction/desorption. Furthermore, we utilized an advanced statistical method, response surface methodology (RSM), to systematically investigate the simultaneous effects of various operating parameters on the optimized catalyst’s performance. Finally, we thoroughly evaluate the catalyst’s long-term stability to ensure sustained and efficient production of H_2_ and CO, underscoring the practical applicability and robustness of our cutting-edge catalyst design.

## 2. Materials and Methods

### 2.1. Materials

The following materials were used: artificial molecular sieves (CBV3024E, CP810B, CBV 20A, and CBV10A from Daiichi Kigenso Kagaku Kogyo Co., Ltd., Osaka, Japan), Ni (NO_3_)_2_.6H_2_O (98%, Alfa Aesar; Thermo Fisher Scientific, Lancashire, UK), and BVT. According to the specifications provided by the manufacturer, the following molecular sieve surfaces and SiO_2_/Al_2_O_3_ ratios are specified: CBV3024E (SiO_2_/Al_2_O_3_ = 30; S_BET_ = 405 m^2^/g), CP810B (SiO_2_/Al_2_O_3_ = 25; S_BET_ = 682 m^2^/g), CBV10A (SiO_2_/Al_2_O_3_ = 13; S_BET_ = 425 m^2^/g), and CBV 20A (SiO_2_/Al_2_O_3_ = 20; S_BET_ = 500 m^2^/g).

### 2.2. Catalyst Preparation

The synthetic molecular sieves (CBV3024E, CP810B, CBV 10A, CBV 20A) were made using a traditional wet impregnation (WI) technique; each had a 5 wt.% metal loading of nickel nitrate hexahydrate (Ni (NO_3_)_2_·6H_2_O, Alfa Aesar; Thermo Fisher Scientific, Lancashire, UK) catalysts. The calcined molecular sieves were gradually treated with a water-based solution of metal precursors. The slurry was made by adding support to the solution while stirring at 80 °C for 2 h, followed by drying at 120 °C overnight, and then calcining for three hours at 600 °C. The catalysts are identified as follows: 5Ni/CBV 3024E, 5Ni/CP810B, 5Ni/CBV 10A, and 5Ni/CBV 20A.

### 2.3. Catalyst Performance Evaluation

An apparatus made by PID Eng & Tech, with dimensions of 9.1 mm in diameter and 30 cm in length, is used to conduct the DRM reaction experiment with 0.1 g of catalyst. Encircling the reactor is a furnace that generates heat. Located axially in the catalyst bed’s center, a K-type thermocouple measures the bed’s temperature. The catalyst must be reduced for one hour at 600 °C in a stream of flowing hydrogen gas (at a flow rate of 30 mL/min) before it can be used. Using a combination of CH_4_, CO_2_, and N_2_ at flow rates of 30 mL/min, 30 mL/min, and 10 mL/min, the DRM reaction was conducted over a reduced catalyst at 700 °C and atmosphere pressure of 1. An online gas chromatography system, which includes a thermal conductivity detector (TCD), Porapak Q columns, and a molecular sieve 5A, is used to examine the gas streams entering and leaving the system. The following is the formula for determining the H_2_ yield and CO yield:

(1) $$H_{2}\;\mathrm{yield}\;(\%)=\frac{Mole\;of\;H_{2}\;in\;Product}{2\times Mol\;of\;{{CH}_{4}}_{in}}\times 100\%$$

(2) $$\mathrm{CO}\;\mathrm{yield}\;(\%)=\frac{Mole\;of\;CO\;in\;Product\;}{Mol\;of\;{{CH}_{4}}_{in}+Mol\;of\;{{CO}_{2}}_{in}}\times 100\%$$

(3) $$\frac{H_{2}}{CO}=\frac{Mole\;of\;H_{2}\;Produced\;}{Mole\;of\;CO\;Produced}$$

### 2.4. Systematic Testing for Improvement

In this study, central composite design (CCD) (Stat-Ease Design Expert software, version 13) was employed with three independent variables that included reaction temperature (A), CH_4_/CO_2_ ratio (B), and space velocity (SV) (C) to predict the optimum process conditions for the dry reforming of CH_4_ over the optimum-obtained 5Ni/CBV3024E during the 300 min on stream. Fifteen trials were conducted, three times each, as part of the experimental design and optimization.

### 2.5. Catalyst Characterization

The Rigaku (Miniflex) diffractometer, operating at 40 mA and 40 kV, was used to perform the powder X-ray diffraction (XRD) investigation of the newly prepared catalyst using Cu Kα radiation (λ = 0.15406 nm). A laser Raman spectrometer from JASCO in Japan was used for the Raman analysis. It had a 532 nm beam excitation and 1.6 mW laser intensity. The sample was exposed for 10 s at 3 accumulations. Quantification of graphitization and carbon deposition type on catalysts was completed with the use of a laser Raman spectrometer (NMR-4500-JASCO, Tokyo, Japan) manufactured by JASCO. A 532 nm wavelength excitation laser was utilized. The IR Prestige-21 SHMADZU was used to record the Fourier transform infrared (FTIR) spectra. Surface characteristics and functional groupings of the samples were investigated using the measures. To conduct the H_2_-TPR, a 0.07 g sample was heated to 1000 °C at a rate of 10 °C/min while being cycled through a gas mixture of H_2_/Ar (*v*/*v*, 10/90) at a rate of 40 mL/min. A thermal conductivity detector was used to monitor the signal of H_2_ consumption. Carbon dioxide (CO_2_) temperature-programmed desorption was used to examine the decreased catalyst’s basicity using the same detector. At room temperature, a mixture of 10% CO_2_/He gas was allowed to interact with 70 mg of catalyst. Various strengths of CO_2_ adsorption over the catalyst were based on the strengths of the basic sites. Depending on the CO₂ binding strength to the surface, desorbed CO_2_ temperatures were different when the temperature was raised to 1000 °C. Using TCD, the amount of CO₂ that was adsorbed was analyzed. This cycle of H_2_TPR-CO_2_TPD-H_2_ TPR persisted constantly.

### 2.6. Determinations of Porosity Parameters

The specific surface area (SSA), pore volume, and average pore diameter were determined using nitrogen adsorption–desorption isotherms at liquid N2 temperature (77 K) on a Micromeritics TriStar II plus instrument. The BET equation P/[(Vm − V) P0] = 1[(C − 1)Vm] + (C − 1)/[C(Vm − V)P0] was employed to calculate SSA, where P = the equilibrium pressure of the gas; P0 = is the saturation pressure of the gas; V = is the volume of gas adsorbed; Vm = is the volume of gas corresponding to a complete monolayer coverage of the adsorbent; C = is a constant related to the energy of adsorption. Pore volume was calculated by subtracting the monolayer volume from the total adsorbed gas volume at a relative pressure of approximately 0.99. The average pore diameter was derived from the total pore volume and specific surface area using the relation Dpore = 4 Vpore/S. The pore size distribution was determined using the BJH method from the desorption branch of the isotherm. Before analysis, samples were degassed at 300 °C under vacuum for 24 h to remove any adsorbed contaminants.

## 3. Results and Discussion

### 3.1. Characterization Results

**Figure 2:**
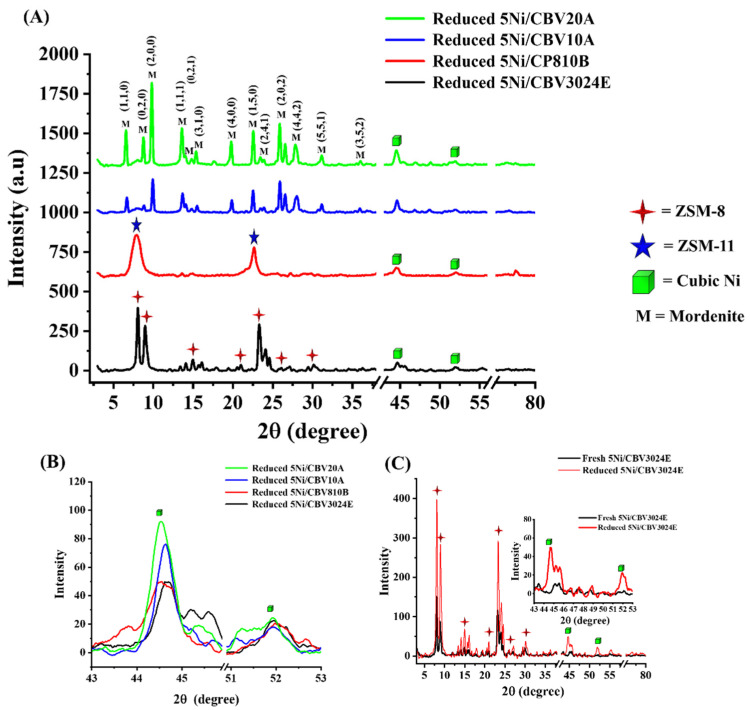
X-ray diffraction of reduced-5Ni/CBV3024E, reduced-5Ni/CP810B, reduced-5Ni/CBV10A, and reduced-5Ni/CBV20A (**A**) from 0 to 80° Bragg’s angle, (**B**) from 43 to 53° Bragg’s angle. (**C**) X-ray diffraction pattern for fresh and reduced Ni/CBV3024E.

The X-ray diffraction study of reduced-5Ni/CBV3024E, reduced-5Ni/CP810B, reduced-5Ni/CBV10A, and reduced-5Ni/CBV20A catalysts is shown in Figure 2A. The reduced-5Ni/CBV20A catalysts have the typical characteristic diffraction pattern of mordenite at Bragg’s angle 6.57°, 8.7°, 9.8°, 13.6°, 14.0°, 14.8°, 15.36°, 19.7°, 22.56°, 23.46°, 23.87°, 25.89°, 26.56°, 27.83°, 31°, 35.47°, 36°, 44.5°, 47°, and 48.8°, which corresponds to following planes: (1,1,0), (0,2,0), (2,0,0), (1,1,1), (1,3,0), (0,2,1), (3,1,0), (4,0,0), (1,5,0), (2,4,1), (0,0,2), (2,0,2), (3,5,0), (4,4,2), (5,1,1), (2,4,2), (3,5,2), (7,3,2), (9,1,1), and (5,9,1), respectively [27]. The XRD diffraction pattern of reduced-5Ni/CBV10A and reduced-5Ni/CBV20 catalysts are the same but the crystalline peak intensity of the former is less than the latter one. It indicates less crystallinity in the case of reduced-5Ni/CBV10A catalyst than reduced-5Ni/CBV20A. Reduced-5Ni/CP810B catalyst shows the typical diffraction pattern of ZSM-11 at Bragg’s angle 7.85° and 22.7° (JCPDS reference number 00-038-0248), whereas reduced-5Ni/CBV3024E catalyst is recognized as ZSM-8 having diffraction patterns about 8°, 9.0°, 13.4°, 14°, 14.85°, 15.6°, 16°, 17.9°, 19.3°, 20.7°, 23.3°, 24°, 24.6°, 25.9°, and 29.9° Bragg’s angle (JCPDS reference number 00-041-0411), respectively [28]. As all catalysts are reduced, so the diffraction pattern of the metallic cubic Ni phase is observed at 44.5°, 52° Bragg’s angle (JCPDS reference number 96-210-0650). Table 1 displays the crystallinity sizes of Ni over reduced catalysts. The crystalline sizes of metallic Ni for reduced-5Ni/CBV3024E, reduced-5Ni/CBV810B, reduced-5Ni/CBV10A, and reduced-5Ni/CBV20A are calculated as 13.9 nm, 13.9 nm, 15.9 nm, and 22.2 nm, respectively (Table 1 and Figure 2B). The different crystalline sizes of metallic Ni are due to the dispersion of active sites over molecular sieves (having different compositions of silica–alumina) as well as pores constraints given by molecular sieves. It is noticeable that the crystalline peak intensity of the 5Ni/CBV3024E, 5Ni/CP810B, and 5Ni/CBV20A catalysts are sharpened upon reduction, whereas a reverse trend is observed for 5Ni/CBV10A (Figure 2 and Figure S1). Thermal-assisted reduction under H_2_ brings depletion of surface hydroxyl over the catalyst surface. finite temperature, thermal fluctuations, and the free energy function are key factors of crystalline growth [29]. During thermal-assisted reduction under H_2_, a finite temperature of 600 °C is set. However, at the crystalline nucleation sites, there may be thermal fluctuation due to variations in thermal conductivity at different silica–aluminate materials (molecular sieve). Thermal fluctuation allows atoms to wander between alternative positions, which may harm crystallite growth. Again, the lower free energy at the crystalline nucleation site welcomes the atoms to join the crystallite growth process.

**Figure 3:**
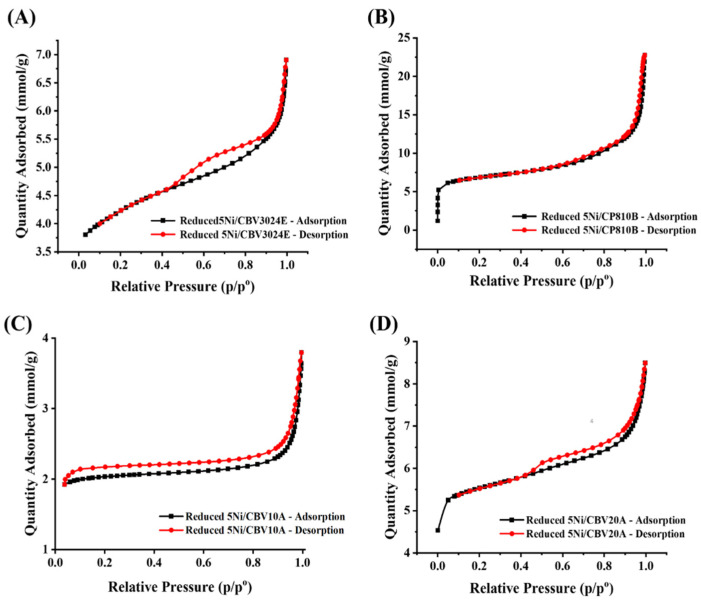
N_2_ adsorption–desorption profile of (**A**) reduced-5Ni/CBV3024E, (**B**) reduced-5Ni/CP810B, (**C**) reduced-5Ni/CBV10A, (**D**) reduced-5Ni/CBV20A.

The surface area and porosity result of reduced-5Ni/CBV3024E, reduced-5Ni/CP810B, reduced-5Ni/CBV10A, and reduced-5Ni/CBV20A catalysts are shown in Figure 3 and Table 2. The adsorption isotherm of all catalysts belongs to type IV, having an H3 hysteresis loop, which is characterized by infinite adsorption at a high relative pressure (p/p°), indicating the presence of slit-like pores [30]. The surface area of the Ni-incorporated molecular sieve is significantly less than the pristine molecular sieve, indicating the deposition of Ni crystallite into the pores of the sieve. The forced closure along the desorption branch is observed at different relative pressures for each catalyst [31]. This is known as the tensile strength effect. The closure point at a certain relative pressure is due to the desorption of adsorbate from pores at which stress in the adsorbate reaches tensile strength [32]. Reduced-5Ni/CBV10A catalyst belongs to a mordenite porous framework having a minimum SiO_2_/Al_2_O_3_ ratio (13), minimum surface area (142 m^2^/g), and pore volume (0.06 cm^3^/g). Such inferior porous architecture in 5Ni/CBV10A results in an early closure point of the desorption curve (at 0.04 p/p°). 5Ni/CBV20A catalyst again belongs to the mordenite framework, having a higher SiO_2_/Al_2_O_3_ ratio (20). The surface parameter of reduced-5Ni/CBV20A is improved two times more (390 m^2^/g surface area and 0.13 cm^3^/g pore volume) than reduced-5Ni/CBV10A. Reduced-5Ni/CP810B catalyst has a ZSM-11-type porous framework, having a higher SiO_2_/Al_2_O_3_ ratio (25), but the most enriched surface parameters like surface area (496 m^2^/g) and pore volume (0.62 cm^3^/g). Reduced-5Ni/CBV3024E catalyst comprises the highest ratio of silica/alumina (30), and its pore architect belongs to the ZSM-8 framework. It attains two times the surface area (304 m^2^/g) and pore volume (0.13 cm^3^/g) of the reduced-5Ni/CBV10A catalyst. The closure point for reduced-5Ni/CBV20A and reduced-5Ni/CBV3024 catalyst falls between 0.41 and 0.42 p/p°. The pore size distribution plot (dV/dlog(w) vs. W; where “V” and “w” are pore volume and pore width) shows the presence of multimodal pores from the microporous-to-macroporous range (Figure S2). However, the average pore diameter of supports and catalysts vary from 7.52 to 16.58 nm and 5.55 to 15.70 nm in the mesoporous range, respectively.

**Figure 4:**
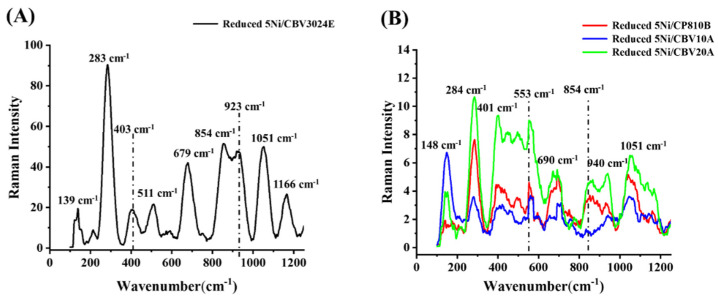
Raman spectra of (**A**) reduced-5Ni/CBV3024E, (**B**) reduced-5Ni/CBV810B, reduced-5Ni/CBV10A, and reduced-5Ni/CBV20A.

The Raman spectra of reduced-5Ni/CBV 3024E, reduced-5Ni/CBV810B, reduced-5Ni/CBV 10A, and reduced-5Ni/CBV 20A catalysts are shown in Figure 4. Interestingly, upon reduction, the intensity of the Raman spectra of reduced-5Ni/CBV3024E catalyst is increased prominently, whereas the rest of the catalysts’ selected vibration modes of Raman spectra are slightly intensified upon reduction (Figure S3). That means the polarizability pattern over the catalyst is modified greatly upon reduction under hydrogen and it is specified as the aluminosilicates being the architect of molecular sieve. The Raman vibration bands corresponding to the bending and starching vibration modes of O-Si-O and Si-O have been reliably identified at approximately 511 cm^−1^ [33] and 854 cm^−1^ [34]. Cubic alumina is itself Raman, inactive [35], but it perturbs the stretching vibrations of silicates under the alumino–silicate framework, which results in a series of Raman bands for vibration of Si-O at 283 cm^−1^, 403 cm^−1^, 923 cm^−1^, 1051 cm^−1^, and 1166 cm^−1^ and vibration bands for Al-O at 679 cm^−1^ [36,37]. The Raman vibrational frequency at 139 cm^−1^ is attributed to the translation of Si/Si and Al/Al lattice modes [35,38]. The additional Raman bands at 446 cm^−1^ and 553 cm^−1^ are attributed to ring vibration in the zeolite framework [39]. The reduced-5Ni/CBV 3024E catalyst has the highest Si/Al ratio so it shows the most intense Raman bands. In the same way, reduced-5Ni/CBV10A catalyst has a minimum Si/Al ratio and so it has minimum Raman band intensity for Si-O and Al-O vibration. The other two catalysts, reduced-5Ni/CP810B and reduced-5Ni/CBV20A, have comparable Raman bands (Figure 4B).

**Figure 5:**
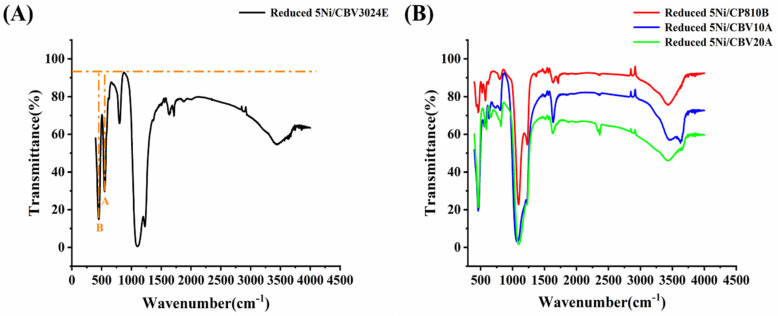
FTIR spectra of (**A**) reduced-5Ni/CBV3024E, (**B**) reduced-5Ni/CP810B, reduced-5Ni/CBV10A, and reduced-5Ni/CBV20A.

The infrared spectra of reduced-5Ni/CBV3024E, reduced-5Ni/CP810B, reduced-5Ni/CBV10A, and reduced-5Ni/CBV20A catalysts are shown in Figure 5. The molecular sieve CBV3024E is of the ZSM type, which may be identified by the presence of an infrared band with a wavelength of approximately 548 cm^−1^ (Band A) and 448 cm^−1^ (Band B) [40]. The intensity ratio of the peak at 548 cm^−1^ (I_A_) and 448 cm^−1^ (I_B_) for the reduced-5Ni/CBV3024E catalyst is found to be 0.8. The I_A_/I_B_ > 0.6 is characterized as ZSM-8 zeolite [28] (Figure 5A). In the reduced-5Ni/CP810B catalyst, the peak at 548 cm^−1^ is split into two peaks at 528 cm^−1^ and 572 cm^−1^. Here, the vibration peak intensity at 528 cm^−1^ is selected as Band A. The I_A_/I_B_ ratio for the reduced-5Ni/CP810B catalyst is found to be 0.28. I_A_/I_B_ < 6 is the characteristic ratio for ZSM-11 among the ZSM family. Reduced-5Ni/CBV10A and reduced-5Ni/CBV20A exhibit typical vibration patterns of mordenite at 460 cm^−1^, 565 cm^−1^, 800 cm^−1^, and 1100 cm^−1^ [41].

**Figure 6:**
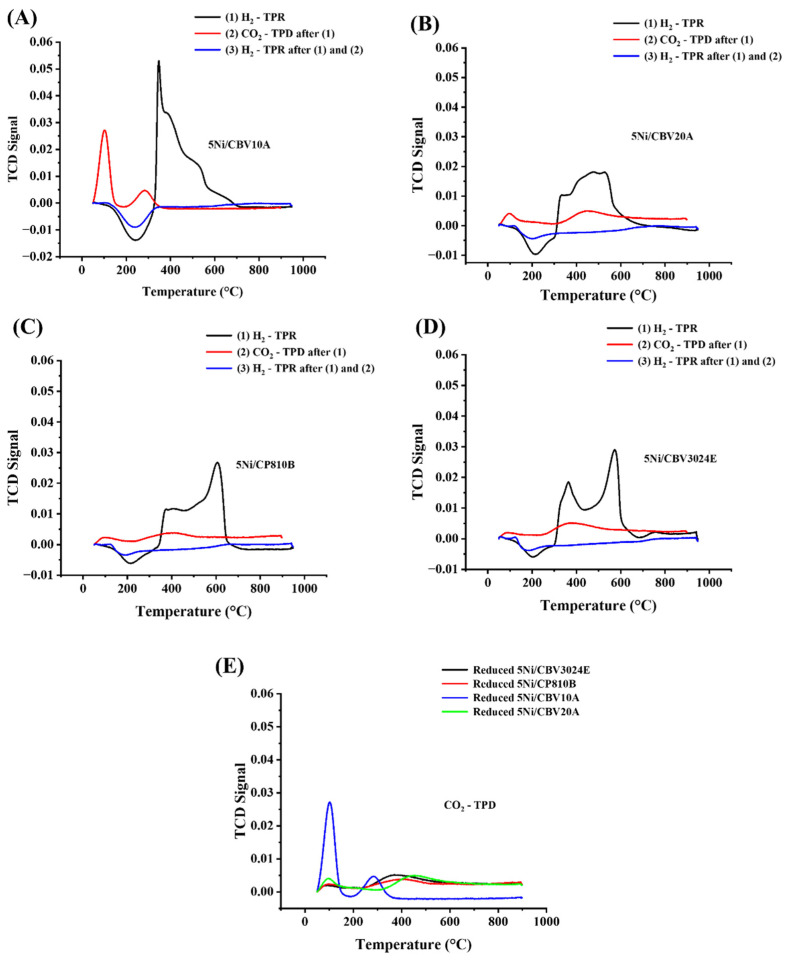
The cyclic H_2_TPR-CO_2_TPd-H_2_TPR study of (**A**) 5Ni/CBV10A, (**B**) 5Ni/CBV20A, (**C**) 5Ni/CP810B, (**D**) 5Ni/CBV3024E, (**E**) CO_2_-TPD of reduced-5Ni/CBV3024E, reduced-5Ni/CP810B, reduced-5Ni/CBV10A, and reduced-5Ni/CBV20A catalyst.

The cyclic H_2_TPR-CO_2_TPD-H_2_TPR results of different Ni-containing molecular sieves are shown in Figure 6 and Table 3. The reduction peak below 300 °C is found to be negative due to hydrogen spillover into pores of Ni-containing molecular sieves [42,43]. The H_2_-TPR profile above 300 °C is due to the consumption of H_2_ by reducible metal oxide over the catalysts. In H_2_-TPR, the 5Ni/CBV10A catalyst absorbs the maximum amount of H_2_ (20 cm^3^/g), indicating the presence of the maximum amount of reducible NiO in the catalyst. Interestingly, H_2_ is consumed mainly at about 350 °C and a little at high temperatures. This peak is attributed to the reduction in Ni^2+^, which is secluded within the charge compensation sites of zeolite [44,45]. The DRM reaction is carried out over a reduced catalyst, and the reduced catalyst may contain basicity for possible the interaction of acidic CO_2_ gas during the DRM reaction. The CO_2_-TPD profile of the reduced catalyst showed that most of the CO_2_ desorption occurs (2.2 cm^3^/g) at about 100 °C, which is typically attributed to weak basic sites or basicity contributed by surface hydroxyl [46]. At about 275–400 °C, there is again desorption of some CO_2_ (0.71 cm^3^/g), which indicates that basic sites of moderate strength (basicity contributed by surface oxide) are also present over the catalyst surface [47,48,49].

Interestingly, after CO_2_ desorption of the reduced catalyst, if H_2_-TPR is again carried out, there is no reduction peak. It indicates that CO_2_ is an oxidizing gas, and it can oxidize the carbon deposit during the DRM, but it will not oxidize the catalytic active sites (metallic Ni). That means active sites remain stable under the oxidizing gas environment during DRM.

The H_2_ consumption over 5Ni/CBV20A and 5Ni/CP810B are comparable (15–15.7 cm^3^/g) and it is about 25% less than 5Ni/CBV10A. So, the amount of reducible NiO species is proportionally less over 5Ni/CBV20A and 5Ni/CP810B catalysts. Among 5Ni/CBV20A and 5Ni/CP810B, a relatively higher amount of H_2_ is consumed at a high-temperature region (600 °C) over 5Ni/CP810B. The reduction peak of NiO in zeolite at about 500 °C was reported for such NiO particles, which were located inside the zeolite channel [44,45,50]. So, here, the reduction peak of about 600 °C can be attributed to the reduction in strong interactions inside the pore channels of the molecular sieve. Overall, it can be said that the amount of reducible species is similar in 5Ni/CP810 and 5Ni/CBV20A, but the earlier one has a greater amount of NiO inside the pore channel of the molecular sieve. The CO_2_-TPD profile of these reduced catalysts shows depletion of low-temperature desorption peaks (concerning 5Ni/CBV10 catalyst), indicating the absence of weak basic sites due to surface hydroxyl. However, the basic sites of moderate strength are present over both catalysts considerably. After CO_2_-TPD treatment of reduced catalyst, if H_2_-TPR is again run, there is no reducible peak. It indicates that the active sites remain stable in the presence of oxidizing gas like CO_2_. The reducibility pattern of 5Ni/CBV3024E is similar to 5Ni/CP810B but it is shifted more towards a relatively lower reduction temperature. That means the 5Ni/CBV3024E catalyst has a higher edge of reducibility than the 5Ni/CP810B catalyst. The basicity pattern of the reduced-5Ni/CBV3024E catalyst is similar to the reduced-5Ni/CP810B catalyst. Here also, the active sites remain stable against oxidizing gas CO_2_ (as observed in the rest of the catalysts).

**Figure 7:**
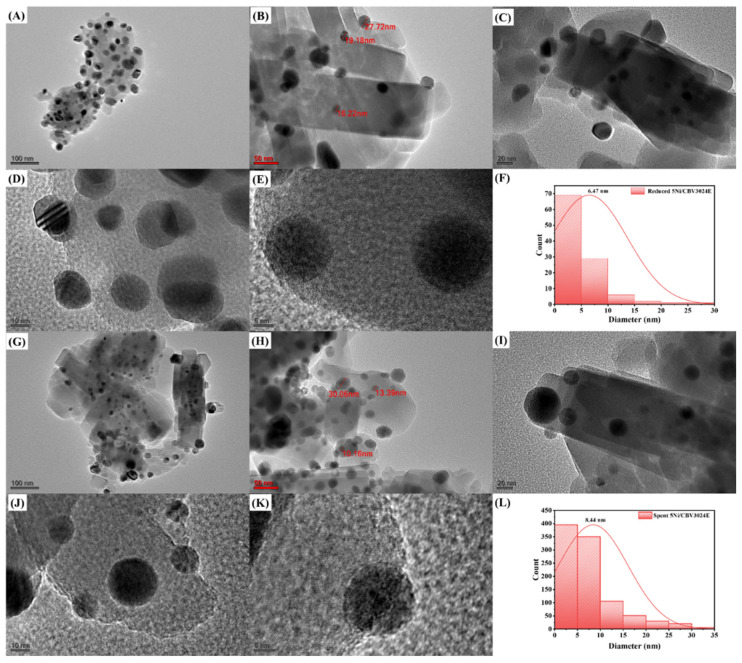
TEM image of reduced-5Ni/CBV3024E at (**A**) 100 nm, (**B**) 50 nm, (**C**) 20 nm, (**D**) 10 nm, (**E**) 5 nm. (**F**) Particle size distribution over reduced-5Ni/CBV3070E. TEM image of spent-5Ni/CBV3070E at (**G**) 100 nm, (**H**) 50 nm, (**I**) 20 nm, (**J**) 10 nm, (**K**) 5 nm. (**L**) Particle size distribution over spent-5Ni/CBV3070E.

The grainy and featureless textures of 5Ni/CBV3024E, 5Ni/CBV20A, and 5Ni/CP810B are evident in SEM images (Figure S4). The TEM image and particle size distribution of reduced and spent 5Ni/CBV3024E catalysts are shown in Figure 7. Analysis of the reduced catalysts using X-ray diffraction (XRD) confirms the conversion of NiO to metallic Ni. TEM images show larger, darker features that could be indicative of larger Ni particles on the catalyst surface. Smaller, fainter contrasts might correspond to smaller particles within the zeolite pores. However, due to TEM contrast limitations, definitive location assignment is difficult. The average particle size of Ni is found at 6.47 nm over a reduced-5Ni/CBV3024E catalyst. In the spent-5Ni/CBV3024E, the average particle size grows to 8.44 nm. Interestingly, no carbon nanostructures like nanofiber and nano-tubes are observed over the catalyst that was used.

### 3.2. Catalytic Activity Results

**Figure 8:**
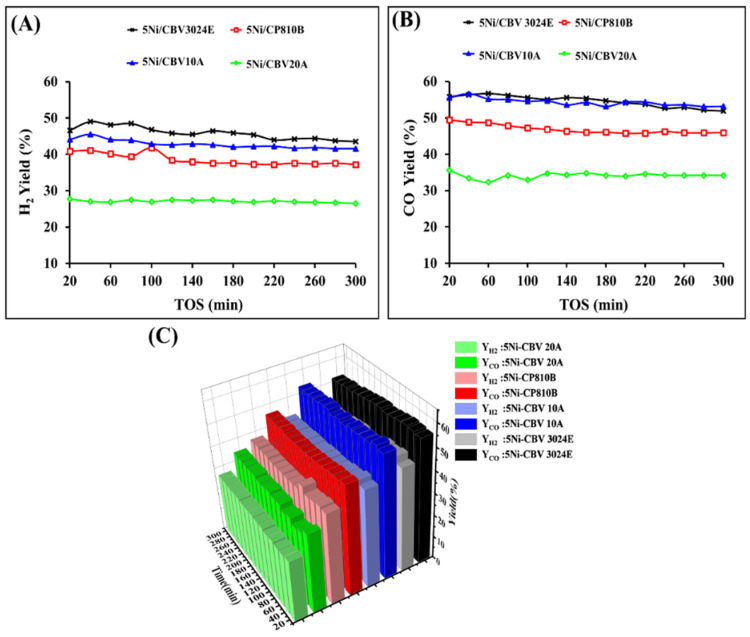
Catalytic activity operated at 700 °C, CH_4_:CO_2_ = 1:1, P = 1 atom, GHSV = 42 L gcat^−1^ h^−1^ (**A**) H_2_ yield vs. TOS, (**B**) CO yield vs. TOS, (**C**) “H_2_ yield and CO yield” vs. TOS.

The catalytic activity results over the different catalysts in terms of H_2_ yield ($Y_{H_{2}}$) and CO yield ($Y_{CO}$) are shown in Figure 8. The catalytic performance over reduced-5Ni/CBV20A catalyst is inferior. It shows 28–27% H_2_ yield and 36–34% CO yield during 300 min on stream. Over the rest of the three catalysts, the H_2_ yield is increased in the following order during 300 min on stream: reduced-5Ni/CP810B ($Y_{H_{2}}$ = 41–37%) < reduced-5Ni/CBV10A ($Y_{H_{2}}$ = 44–42%) < reduced-5Ni/CBV3070E ($Y_{H_{2}}$ = 47–44%). The CO yields over 5Ni/CP810B, 5Ni/CBV3070E, and 5Ni/CBV10A are observed to be 46%, 52%, and 53% at the end of 300 min.

### 3.3. Discussion

Ni containing different molecular sieves are investigated for DRM reforming of methane. XRD, Raman, and IR spectra of reduced catalyst samples confirm that CBV10A and CBV20A belong to mordenite-based support, whereas CP810B and CBV3024E belong to ZSM-11 and ZSM-8-based supports, respectively. The silica–alumina ratios of CBV10A, CBV20A, CP810B, and CBV3024E are 13, 20, 25, and 30, respectively. ZSM-5, ZSM-11, and modernities are categorized under pore channel types. The porous channel of mordenite is made up of 12-ring channels and 8-ring channels where 12-ring channels are connected through 8-ring channels arranged in parallel and perpendicular fashion [51]. ZSM-11 is weaved by intersectional straight 12-ring channels, whereas ZSM-8 has intersectional straight and sinusoidal 12-ring channels [52].

CBV20A and CP810B contain mordenite and ZSM-11-type pores channels. Interestingly, upon impregnating 5 wt.% Ni over this molecular sieve, NiO is dispersed over the outer surface of the molecular sieve (at charge compensation sites) and inside the pore channel of the molecular sieves (confirmed by H_2_ TPR and TEM). Approximately the same amount of reducible NiO is reduced into metallic Ni over these molecular sieves throughout the reduction in catalysts. However, in 5Ni/CP810B, there are more active sites incorporated into the pore channel than the 5Ni/CBV20A catalyst. Clearly, this difference is due to more dispersion of smaller Ni crystallites (13.9 nm) into the 12-ring channels of the 5Ni/CP810B catalyst (ZSM-11 architect). In the case of reduced-5Ni/CBV20A (SiO_2_/Al_2_O_3_ = 20), porous channels are the mordenite type where 12-ring channels are connected through 8-ring channels. The crystallite size of Ni is also the largest at 22.2 nm. So, passing large Ni crystallites into “8-ring channels” or “12-ring channels through 8-ring channels” is relatively difficult. Overall, the reduced-5Ni/CBV20A catalyst has a smaller number of active sites in the channel, whereas the reduced-5Ni/CP810B catalyst has a larger population of active sites in the channel.

Additionally, the reduced-5Ni/CP810B catalyst has the highest surface area and pore volume among all catalysts. The active sites inside the channel are quite stable compared to the active sites on the outer surface of the molecular sieve. Reduced-5Ni/CBV20A catalyst, which has less amount of stable active site into mordenite’s porous architects, shows 27% H_2_ yield constantly and 36–34% CO yield during 300 min on stream. However, reduced-5Ni/CP810B, which has a higher population of stable active sites in the ZSM-11’s porous architects, performs better. Initially, 41% H_2_ yield and 50% CO yield were observed over reduced-5Ni/CP810B, which slowed down to 37% H_2_ yield and 46% CO yield during 300 min on stream, respectively. The presence of higher CO yield than H_2_ yield over both catalysts confirms the existence of hydrogen-consuming reactions like reverse water gas shift reactions as major side reactions (Figure 8C) [50].

**Figure 9:**
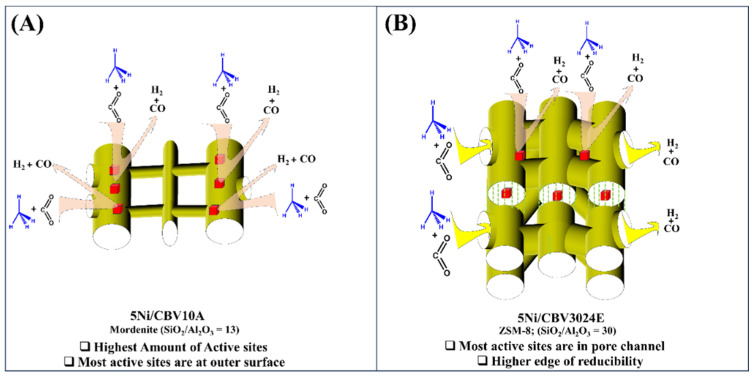
The reaction Schemes of DRM over (**A**) reduced-5Ni/CBV10A catalyst, having mordenite-based porous architecture and (**B**) reduced-5Ni/CBV3024E catalyst, having ZSM-8 based porous architecture.

5Ni/CBV10A catalyst also has a mordenite-based porous framework but a lower silica–alumina ratio (SiO_2_/Al_2_O_3_ = 13). Increasing the proportion of alumina with silica was reported to reduce the size of NiO aggregates, resulting in enhanced reducibility and high dispersion [53]. XRD results also show a smaller Ni crystallite (15.9 nm) over reduced-5Ni/CBV10A than 22 nm Ni crystallite over reduced-5Ni/CBV20A. It indicates a higher dispersion of Ni crystallites over a reduced-5Ni/CBV10A catalyst. 5Ni/CBV10A catalyst has 33% more reducible NiO-species than 5Ni/CBV20A and 5Ni/CP810B catalysts. The minimum surface area and pore volume, early closure point of desorption, and narrower 8-ring channels of the mordenite framework make localization of Ni difficult into the pore channels. The H_2_-TPR result justifies the presence of most of the reducible species at the outer surface of the molecular sieve which is stabilized at the charge compensation sites of the molecular sieve. Despite the deficit of active sites in the pore channels, the reduced-5Ni/CBV10A catalyst acquires the highest amount of total active sites and achieves 42% H_2_ yield and 53% CO yield during the 300 min time on stream. The porous architect of reduced-5Ni/CBV3024E and reduced-5Ni/CP810B are like ZSM-8 and ZSM-11 types. The former has straight intersectional sinusoidal 12-ring channels, but the latter has simply straight intersectional 12-ring channels. The Ni’s crystallinity, reducibility profile, and basicity profile of reduced-5Ni/CBV3024E is very similar to reduced-5Ni/CP810B. But the former has a higher edge of reducibility than the latter. Overall, the catalytic performance of reduced-5Ni/CBV3024E is best and it acquires 44% H_2_ yield and 52% CO yield after up to 300 min on stream. Now, the reaction scheme for DRM over the efficient Ni-supported molecular sieve, one having minimum SiO_2_/Al_2_O_3_ ratio (13) and another having maximum SiO_2_/Al_2_O_3_ ratio (30), can be illustrated (Figure 9). Figure S5 shows the TGA profiles of the used samples after 5 h of reaction. The figure displays that the 5Ni/CBV10A sample exhibits the greatest carbon deposition, whereas the carbon deposit is the least over 5Ni/CBV20A as well as 5NiCBV3024E catalysts.

Reduced-5Ni/CBV10A (SiO_2_/Al_2_O_3_ = 13) has mordenite-type pores architecture, and it acquires the least surface area and pore volume. Over this catalyst, localization of metallic Ni into 12-ring channels connected through narrow 8-ring channels is quite difficult due to space constraints and minimum pore volume. However, the maximum population of active sites has been created at the outer surface of the molecular sieve (Figure 9A), which catalyzes the decomposition of the C-H bond (of CH_4_). The sequential oxidation of decomposed CH_4_ by CO_2_ conveys 42% hydrogen yield after up to 300 min. Reduced-5Ni/CBV3024E (SiO_2_/Al_2_O_3_ = 13) has a ZSM-8-type porous architecture (made up of sinusoidal 12-ring channels) and the smallest Ni crystallites resulting in localization of Ni into the pores mostly (Figure 9B). Despite the low concentration of active sites, the active sites clipped under pore channels of reduced-5Ni/CBV3024E catalyst are quite stable. The active sites (the outer surface as well as inside the pore channels) catalyze C-H decomposition, which further undergoes oxidation under CO_2_. In the TEM image of this catalyst, carbon nanostructures are also not observed. The catalyst achieves 44% H_2_ yield in up to 300 min. This investigation validates the remarkable performance of the 5Ni/CBV3024E catalyst, building on earlier studies (mentioned in Table S1).

### 3.4. Design of Experiment and Optimization

#### 3.4.1. Central Composite Design (CCD)

CCD is a frequently utilized approach in response surface methodology (RSM), aimed at extracting the maximum amount of information about a process from a limited number of experiments. This technique is effective in modeling quadratic effects and determining optimal conditions for process enhancement based on the desired properties of the design and the number of factors considered. This study’s experimental variables include temperature, the ${{CH}_{4}:CO}_{2}$ ratio, and space velocity, each with specified upper and lower limits. The mean value of x_i_ is (${\bar{X}}_{oi}$) and is given in Equation (4),

(4) $${\bar{X}}_{oi}=\frac{x_{i\;max}+x_{i\;min}}{2}$$

which describes the center of the interval of the lower (x_i min_) and upper limits (x_i max_), and the deviation of either limit from the mean is described in Equation (5):

(5) $${∆x}_{i}=\frac{x_{i\;max}-x_{i\;min}}{2}$$

In experimental design, the coordinates (${\bar{X}}_{o1}$, ${\bar{X}}_{o2}$, ${\bar{X}}_{o3}$, …, ${\bar{X}}_{on}$) define the center point of the experiment. This particular set of input variables is generally incorporated to detect curvature in the response surface. The initial values are converted into coded, dimensionless variables Xi within the range of −1 to 1 using the transformation specified in Equation (6).

(6) $$X_{i}=\frac{x_{i}-{\bar{X}}_{oi}}{{∆x}_{i}}$$

where $x_{i}$, represent the original value of the i-th input variable, and ${∆x}_{i}$ signifies the range of values for that variable.

In experimental design, the outcome variable is often expressed as a polynomial equation of several factors. The Taylor Series expansion is used to derive this polynomial equation, approximating the response surface with a mix of linear, quadratic, and occasionally cubic terms. In our experiment, a modified quadratic polynomial is found, and the full quadratic model with three factors in the general form is given by Equation (7):

(7) $$\hat{Y}=\beta_{0}+\sum_{i=1}^{3}\beta_{i}X_{i}+\sum_{i=1}^{2}\sum_{j=i+1}^{3}{\beta_{ij}X}_{i}X_{j}+\sum_{i=1}^{3}{\beta_{ii}X}_{i}^{2}+$$

where, $X_{1},X_{2}$, and $X_{3}$ are the experimental factors in actual or coded values, $\beta_{0}$ is a constant term, $\beta_{i},i=1,2,3$ are the linear terms, $\beta_{ii}$ are the quadratic terms, $\beta_{ij}$, j=1,2,3 are the interaction terms, and ε is the error term, which represents the random variation in $\hat{Y}$ that is not accounted for by the process parameters [39]. Table 4 lists the actual and coded values of the studied factors.

##### Process Modeling and Analysis of Variance

**Figure 10:**
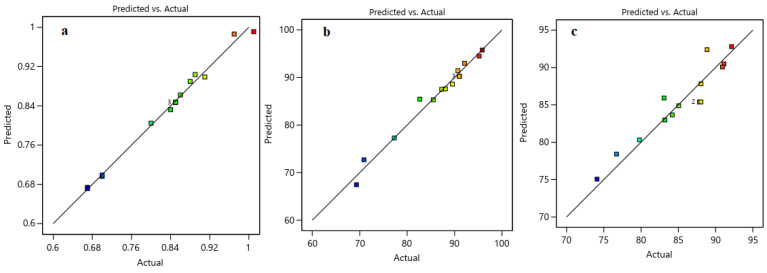
The experimental and the predicted data for (**a**) H_2_/CO, (**b**) H_2_ yield, and (**c**) CO yield.

ANOVA, or analysis of variance, is a statistical technique that partitions the total variability in data into distinct components attributed to various sources. This method is critical in identifying significant factors and their interactions, enhancing a good theoretical model. Process modeling and variance analysis utilize the central composite design (CCD) approach, which optimizes processes, assesses factor interactions, and determines the optimal power transformation for response data to normalize or stabilize variance [40]. Analysis of variance for the various components is shown in Table 5. High F-values and low *p*-values indicate significant model terms at a confidence level of approximately 95%. Moreover, high R^2^ values indicate that the model effectively represents experimental data, as illustrated in Figure 10.

##### Final Equations and Accuracy Models

According to the experimental data, ANOVA was conducted at a significance level α=0.05, and after identifying the significant factor effects while excluding insignificant ones, the following models were developed using Design-Expert software version 13.

(8) $$\hat{H_{2}\;yield}=-554.42115+1.86263\;A-172.05000\;B-0.004409\;C+0.301000\;AB+0.0000051\;AC-0.001332\;A^{2}-59.60000\;{\;B}^{2}$$

(9) $$\hat{CO\;yield}=117.66724-0.031819\;A-3.81000\;B-0.003599\;C+0.0000044\;AC$$

(10) $$\hat{H_{2}/CO}=1.20391-0.001375\;A-0.280110\;B+0.0000018\;C+0.002200\;AB+-0.0000054\;BC-0.514286{\;B}^{2}$$

where A is temperature, B is the feed ratio of reactants, and C is the SV, which is the hourly space velocity.

Within the models (5)–(7), the constant coefficients signify the anticipated value of the response variable when all other factors are at zero. The coefficient of one factor indicates its main effect, illustrating how the response variable changes with a one-unit increase in each factor while holding all other factors constant. The coefficient associated with the interaction of two factors represents the combined effects of these interactions. Positive coefficients indicate a positive or increased influence on the response variable, while negative coefficients indicate the opposite effect.

The coefficient of determination (R^2^) quantifies the proportion of variability in the response variable that is accounted for by the independent variables in the model, serving as a measure of the model’s goodness-of-fit. A high R^2^ value indicates a well-fitting model. The R^2^ values for the expected H_2_/CO, H_2_ yield, and CO yield models are 0.9923, 0.9795, and 0.8846, respectively. These values indicate that the models explain approximately 99.23%, 97.95%, and 88.46% of the total variations in the expected responses.

Table 6 presents the experimental and predicted values of all response variables based on the proposed models. As depicted in Table 6, the proximity of the estimated responses derived from the proposed models to the actual values highlights the models’ capacity to accurately reflect the observed data. This is evidenced by the low absolute error rates of the three models, indicating that, on average, predicted values deviate from actual values by 0.78%, 1.04%, and 1.59%, respectively, as shown in Table 6. A lower Mean Absolute Percentage Error (MAPE) value signifies greater accuracy in the models. In addition, MAPE, Absolute Error Percentage (APE), and Mean Absolute Error (MAE) which are shown in Equations (8)–(10) are used to evaluate the model’s accuracy.

Table 6 confirms a strong correlation between CCD models and experimental results, with R^2^ near 1. This consistency is evident in Figure 10; through plotting predicted against actual values that a crucial for model assessment, we found the close alignment to the X=Y line, indicating a good fit.

(11) $$APE=100\;*\;\frac{\left|E_{i}-P_{i}\right|}{E_{i}}\;\%$$

where APE = Absolute Percentage Error; E_i_: Actual observed value; P_i_: Predicted value from the model.

(12) $$MAE=\frac{1}{n}\sum_{i=1}^{n}\frac{\left|E_{i}-P_{i}\right|}{E_{i}}$$

MAE = Mean Absolute Error; n = Number of data points.

(13) $$MAPE=100\;*\;\frac{1}{n}\sum_{i=1}^{n}\frac{\left|E_{i}-P_{i}\right|}{E_{i}}\;\%$$

MAPE = Mean Absolute Percentage Error.

#### 3.4.2. Simulation of Design-Expert Program

Due to the endothermic nature of the DRM reaction, higher temperatures significantly boosted the yields of both H_2_ and CO. This can be explained by the increased kinetic energy within the system at higher temperatures. This rise in energy effectively accelerates the movement of reactants (mass transfer), leading to a more efficient reaction and, ultimately, greater production of the desired products.

It was observed that a decrease in product yield alongside an increase in the CH_4_/CO_2_ ratio from 0.5 to 1.0. This suggests that excess CH_4_ is decomposing and depositing carbon, leading to lower overall product output. Additionally, the study found that yields of both H_2_ and CO decreased with a higher reactant ratio and increased space velocity. Interestingly, the ratio of H_2_ to CO increased with a higher reactant ratio but decreased with a higher space velocity.

##### One Factor Effect (2D) Plot

**Figure 11:**
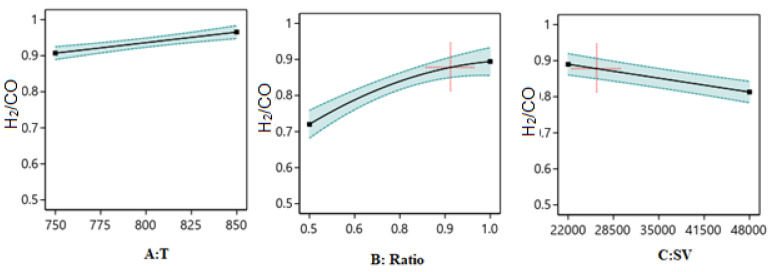
The relationship between the reaction parameters and the H_2_/CO ratios.

**Figure 12:**
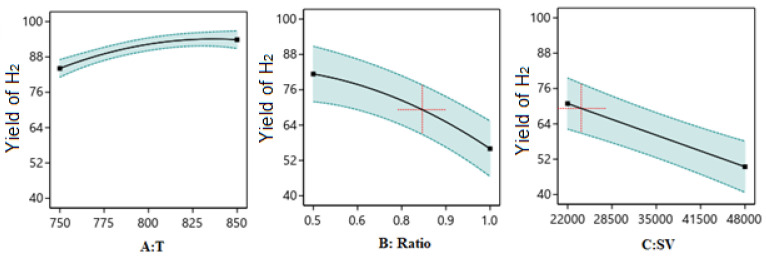
The relationship between the reaction parameters and H_2_ yield percentage.

**Figure 13:**
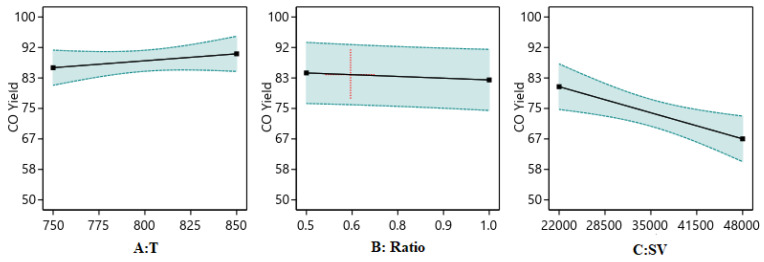
The relationship between the reaction parameters and CO yield percentage.

The effect of each process parameter on the reaction responses is shown in Figure 11, Figure 12 and Figure 13. Figure 11 indicates that increasing temperature, increasing the ratio, and decreasing the SV value will increase H_2_/CO. Figure 12 indicates that increasing temperature, decreasing the ratio, and decreasing the SV value will increase H_2_ yield. Figure 13 indicates that increasing temperature, decreasing the ratio, and decreasing the SV value will increase CO yield.

##### Two Factors Effect (3D Plot)

**Figure 14:**
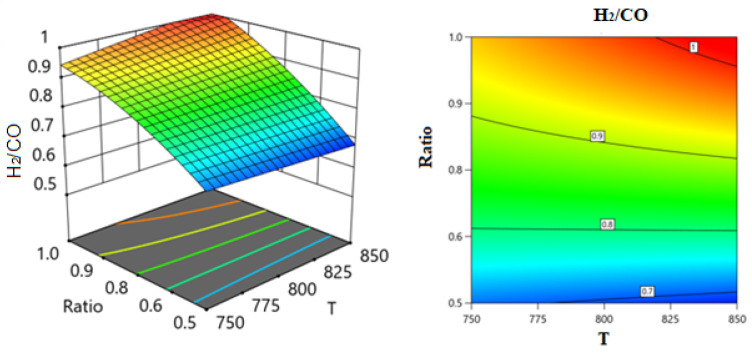
The relationship between the temperature and H_2_/CO ratio at SV = 24,000.

**Figure 15:**
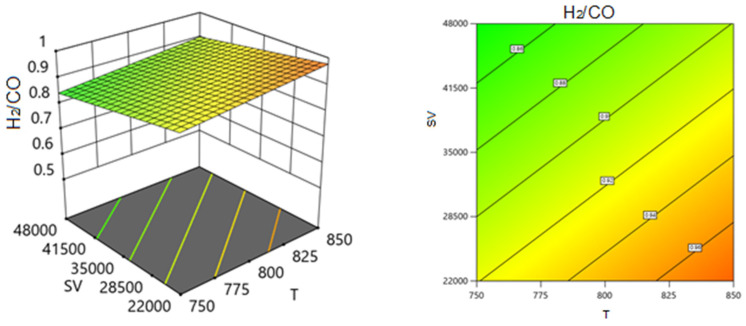
The relationship between the temperature, SV, and H_2_/CO at ratio = 0.89.

**Figure 16:**
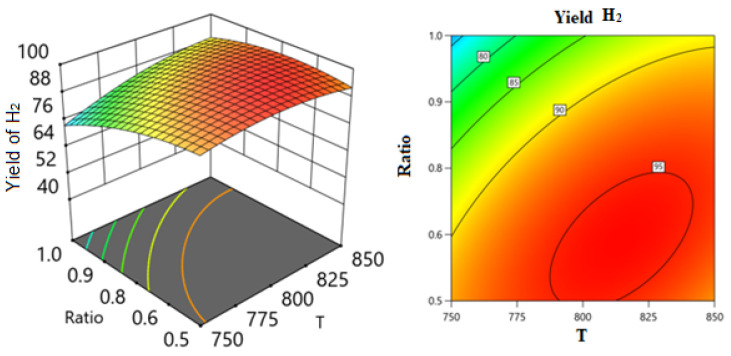
The relationship between the temperature, ratio, and H_2_ yield % at SV = 24,000.

**Figure 17:**
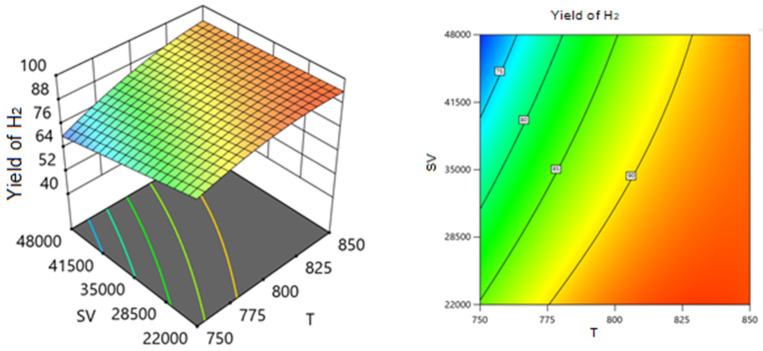
The relationship between the temperature, SV, and H_2_ yield % at ratio = 0.8081.

**Figure 18:**
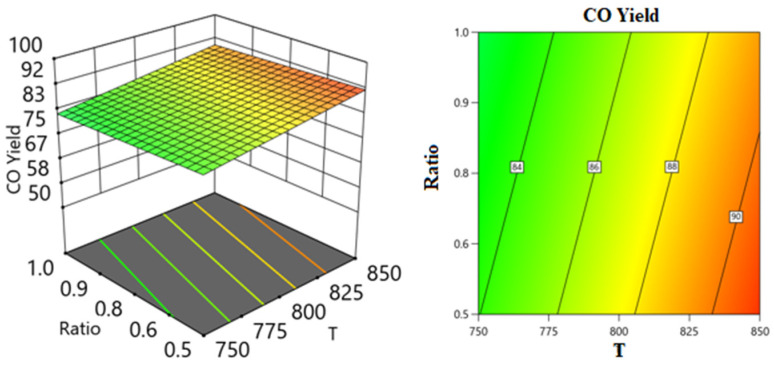
The relationship between the temperature, ratio, and CO yield % at SV = 24,000.

**Figure 19:**
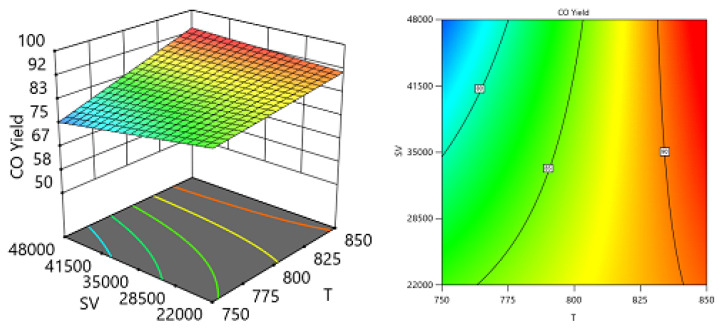
The relationship between the temperature, SV, and CO yield % at Ratio = 0.6209.

Employing the regression coefficients for various components led to the development of a series of response surface methodology (RSM) equations. With the assistance of these equations and the Design-Expert software, response surface plots were generated to predict the conversion or formation of components in the reaction system across two varying process variables while holding the third variable constant. These predictions are illustrated in the 3D models shown in Figure 14, Figure 15, Figure 16, Figure 17, Figure 18 and Figure 19.

Figure 14 and Figure 15 show the three-dimensional response surface plot, which represents the effects of the factors (temperature, SV, and ratio ${{CH}_{4}:CO}_{2}$ on the variation in H_2_/CO. Figure 14 shows the surface plots which represent the relationship between the response variable (H_2_/CO) and the two factors (temperature and the ratio ${{CH}_{4}:CO}_{2}$) at SV = 24,000. It is shown that by increasing the temperature and increasing the ratio ${{CH}_{4}:CO}_{2}$, the H_2_/CO increases. Figure 15 shows the surface plots that represent the functional relationship between a designated response variable (H_2_/CO) and the two-factor variables (Temperature and SV) with ${{CH}_{4}:CO}_{2}$ fixed at 0.89. The response surface shows that with increasing the temperature and decreasing the SV, the H_2_/CO increases. It was observed to increase from 0.67 at 750 °C to 1 at 850 °C.

Figure 16 and Figure 17 show the three-dimensional response surface plots that represents the effects of the factors (temperature, SV, and ratio ${{CH}_{4}:CO}_{2}$) on the variation in the yield of H_2_. Figure 16 shows the surface plots which represent the relationship between the response variable (H_2_ yield) and the two factors, temperature and the ratio ${{CH}_{4}:CO}_{2}$, at SV = 24,000. It is shown that by increasing the temperature and decreasing the ratio, H_2_ yield increases. Figure 17 shows the surface plots that represent the functional relationship between a designated response variable (H_2_ yield) and the two factor variables, temperature and SV, with ${{CH}_{4}:CO}_{2}$ fixed at 0.8081. The response surface shows that by increasing the temperature and decreasing the SV, the yield of H_2_ increases. It was observed to increase from 69.31% at 750 °C to 95.85% at 850 °C.

Figure 18 and Figure 19 show the three-dimensional response surface plots that represents the effects of the factors (temperature, SV, and ratio ${{CH}_{4}:CO}_{2}$) on the variation in CO yields. Figure 18 shows the surface plots which represent the relationship between the response variable (CO yield) and the two factors, temperature and the ratio ${{CH}_{4}:CO}_{2}$, at SV = 24,000. It is shown that with increasing temperature and decreasing ratio, the CO yield increases. Figure 19 shows the surface plots that represent the relationship between the response variable CO yield and the two factors variables, temperature and SV, with ${{CH}_{4}:CO}_{2}$ fixed at 0.6209. The plot shows that with increasing temperature and decreasing SV, the CO yield increases. It was observed to increase from 74.09% at 750 °C to 92.15% at 850 °C.

##### Optimization

**Figure 20:**
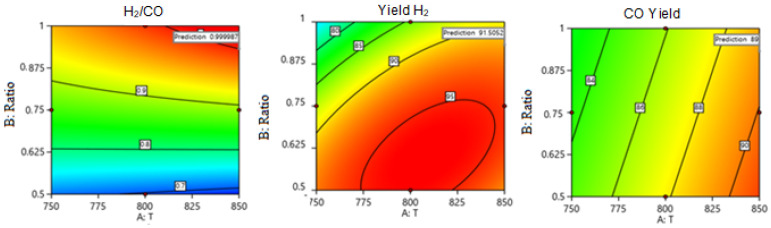
Optimum predicted simultaneous values of H_2_/CO ratio, H_2_ yield, and CO yield at the controlled conditions in Table 7.

The objective of this part is to maximize the production of (H_2_/CO), H_2_ yield, and CO yield at optimum values of factors. Table 6 presents the comparison between theoretical and experimental results, focusing on maximizing H_2_/CO ratio, H_2_ yield percentage, and CO yield percentage under specified conditions and variables (factors). Table 7 compares theoretical and experimental results under the same conditions of temperature (T), methane to carbon dioxide ratio (CH_4_:CO_2_), and space velocity (SV). Figure 20 displays the optimum predicted simultaneous values of H_2_/CO ratio, H_2_ yield, and CO yield at the controlled conditions in Table 7. The comparison shows that the experimental values closely approximate the theoretical predictions, demonstrating consistency and alignment between the expected and experimental outcomes under controlled conditions. The catalytic activity of 5Ni/CBV3024E catalyst towards DRM reaction is compared with previously reported catalyst systems (Table 8) [50,54,55,56,57,58,59,60,61,62,63,64,65,66,67,68,69,70,71,72,73]. At optimized reaction conditions, the H_2_ yield over 5Ni/CBV3024E catalyst ($Y_{H_{2}}$ ≥ 90%) is found to be even higher than NiPd-SiO_2_-Imp catalyst ($Y_{H_{2}}=88\%$) [56]. The current catalyst 5Ni/CBV3024E is free from noble metals but attains a high H_2_ yield at optimized reaction conditions.

## 4. Conclusions

Ni containing different molecular sieves, named CBV10A, CBV20A, CP810B, and CBV3024, are investigated for DRM reaction. Reduced-5Ni/CBV10A catalyst has the lowest silica–alumina ratio and a mordenite-type porous framework, exhibiting a unique behavior. Due to its minimal pore volume, early desorption closure point, and narrow pore channels (8-ring), the Ni active sites are primarily localized on the outer surface, achieving a 42% hydrogen yield over 300 min of continuous operation.

In contrast, the other catalysts, namely, reduced-5Ni/CBV20A, reduced-5Ni/CP810B, and reduced-5Ni/CBV3024E, displayed 25% fewer total active sites, which were distributed both on the outer surface and within the pore channels. Despite reduced-5Ni/CBV20A having double the surface area and pore volume compared to reduced-5Ni/CBV10A, its larger Ni crystallites and narrower 8-ring channels restricted the localization of active sites within the pores. This resulted in a lower hydrogen yield of 27%, as the total number of active sites was inferior.

The reduced-5Ni/CP810B catalyst, featuring broader 12-ring channels of the ZSM-11 type and the smallest Ni crystallites, allowed for more stable active sites within the pores, achieving a better hydrogen yield of 41%. Similarly, the reduced-5Ni/CBV3024E catalyst, with its sinusoidal 12-ring channels and highest silica–alumina ratio, exhibited similar reducibility and crystallinity profiles to reduced-5Ni/CP810B. However, the active sites on reduced-5Ni/CBV3024E formed at relatively lower temperatures, leading to the highest hydrogen yield of 44% over 300 min.

To optimize the process parameters, we used a simulation of the Design-Expert program based on the CCD to develop a quadratic model for RSM and to optimize the process parameters. The ANOVA table obtained suggested that the model was significant. A set of 15 experiments was carried out to study optimization at different reaction conditions. The findings revealed that the reaction temperature had the greatest impact on the process, followed by the space velocity, while the feed ratio had little impact. It was discovered that the ideal parameters for maximizing the response variables were a reaction temperature of 850 °C, a space velocity of 22,000.009 ccg^−1^h^−1^, and a feed ratio of 0.932. Under these conditions, the projected H_2_ and CO yields were 91.5% and 89.37%, respectively, and the H_2_/CO ratio was 1.00. The experimental results obtained under the predicted optimal operating conditions matched the predicted values quite well, and hence, the validation experiment confirmed the model’s accuracy with negligible differences between predicted and observed results.

## Figures and Tables

**Table 1:** The crystallite sizes of the different zeolite-supported catalysts.

Catalyst Name	The Crystallite Size of Ni (nm)
5Ni/CBV 3024E	13.9
5Ni/CBV810B	13.9
5Ni/CBV10A	15.9
5Ni/CBV20A	22.2

**Table 2:** The surface area, pore volume, and average pore size of different reduced catalysts and their corresponding supports.

Reduced Catalyst	Surface Area (m^2^/g)	Pore Volume (cm^3^/g)	Average Pore Diameter (nm)
CBV 3024E	405	0.12	7.52
5Ni/CBV 3024E	304	0.13	5.55
CP810B	682	0.61	14.18
5Ni/CP810B	496	0.62	13.7
CBV 10A	425	0.07	16.58
5Ni/CBV 10A	142	0.06	15.70
CBV 20A	500	0.13	7.82
5Ni/CBV 20A	390	0.13	7.24

**Table 3:** The amount of H_2_ consumption and CO_2_ desorption against different temperatures under H_2_TPR-CO_2_TPD-H_2_TPR cyclic experiments.

	Catalyst Name	Temp. (°C) (Peak 1) 300–400 °C	H_2_ Consumption Amount (cm^3^/g)	Temp. (°C) (Peak 2) 500–600 °C	H_2_ Consumption Amount (cm^3^/g)	Total Amount (cm^3^/g)
**First H_2_-TPR**	5Ni/CBV3024E	363	4.799	572	7.992	12.791
	5Ni/CP810B	405	4.261	606	11.429	15.69
	5Ni/CBV10A	347	20.498	-	-	20.498
	5Ni/CBV20A	477	9.726	525	5.245	14.971
	**Catalyst Name**	**Temp. (°C)** **(Peak 1)** **~100 °C**	**CO_2_ Desorption Amount** **(cm^3^/g)**	**Temp. (°C)** **(Peak 2) ~300–400 °C**	**CO_2_ Desorption Amount** **(cm^3^/g)**	**Total**
**CO_2_-TPD of reduced catalyst**	Reduced-5Ni/CBV3024E	85	0.126	371	0.778	0.904
	Reduced-5Ni/CP810B	96	0.198	383	0.548	0.746
	Reduced-5Ni/CBV10A	103	2.191	283	0.708	2.899
	Reduced-5Ni/CBV20A	97	0.487	443	0.933	1.42

**Table 4:** Actual and coded values for the process parameters.

	Levels	
Process Parameter	−1 (low)	+1 (high)
Gas hour space velocity (ccg^−1^h^−1^)	22,000	48,000
Temperature (°C)	750	850
Ratio ${CH}_{4}:{CO}_{2}$	0.5	1

**Table 5:** Analysis of variance for the various components.

Source	Sum of Squares	df	Mean Square	F-Value	*p*-Value
**Response 1: H_2_/CO ratio (R^2^ = 0.9923)**					
**Model**	0.1514	6	0.0252	172.89	<0.0001
A: Temperature	0.0002	1	0.0002	1.64	0.2367
B: Ratio	0.0099	1	0.0099	67.5	<0.0001
C: SV	0.0008	1	0.0008	5.23	0.0515
AB	0.003	1	0.003	20.72	0.0019
BC	0.0012	1	0.0012	8.39	0.02
	0.0039	1	0.0039	26.42	0.0009
B^2^	0.1514	6	0.0252	172.89	<0.0001
**Response 2: H_2_ yield (R^2^ = 0.9795)**					
**Model**	905.55	7	129.36	47.71	<0.0001
A: Temperature	29.34	1	29.34	10.82	0.0133
B: Ratio	24.95	1	24.95	9.2	0.019
C: SV	102.39	1	102.39	37.76	0.0005
AB	56.63	1	56.63	20.88	0.0026
AC	44.29	1	44.29	16.33	0.0049
A^2^	41.19	1	41.19	15.19	0.0059
B^2^	51.54	1	51.54	19.01	0.0033
**Response 3: CO yield (R^2^ = 0.8846)**					
**Model**	348.01	4	87	19.16	0.0001
A: Temperature	5.45	1	5.45	1.2	0.2989
B: Ratio	7.26	1	7.26	1.6	0.2347
C: SV	45.25	1	45.25	9.97	0.0102
AC	32.09	1	32.09	7.07	0.0239

Where A represents the temperature, B is the CH_4_/O_2_ ratio, and C represents the space velocity.

**Table 6:** Experimental and predicted data results for various components of the reaction system.

Exp No	Variables			Response								
	A: Temperature	B: CH_4_/CO Ratio	C: SV	H_2_/CO			H_2_ Yield			CO Yield		
				Exp.	Pred.	$\%\left|Error\right|$	Exp.	Pred.	$\%\left|Error\right|$	Exp.	Pred.	$\%\left|Error\right|$
**1**	**800**	**0.75**	35000	0.85	0.8471	0.34	91.01	90.26	0.82	87.84	85.4	2.78
2	800	0.75	35000	0.85	0.8471	0.34	91.01	90.26	0.82	87.84	85.4	2.78
3	800	1	48000	0.91	0.8988	1.23	77.29	77.3	0.01	83.2	82.97	0.27
4	750	1	35000	0.89	0.9038	1.55	69.31	67.46	2.67	76.71	78.41	2.22
5	850	1	35000	0.97	0.9863	1.68	89.55	88.62	1.04	91.13	90.48	0.71
6	800	0.75	35000	0.85	0.8471	0.34	91.01	90.26	0.82	88.04	85.4	3.00
7	800	1	22000	1.01	0.9913	1.85	82.68	85.45	3.35	83.09	85.91	3.39
8	750	0.75	48000	0.8	0.8046	0.58	70.9	72.71	2.55	74.09	75.06	1.31
9	800	0.5	48000	0.67	0.6738	0.57	88.07	87.62	0.51	85.05	84.88	0.20
10	750	0.75	22000	0.86	0.8621	0.24	87.28	87.52	0.27	84.19	83.66	0.62
11	800	0.5	22000	0.7	0.6963	0.53	95.85	95.78	0.08	88.05	87.82	0.26
12	750	0.5	35000	0.7	0.6987	0.17	85.51	85.31	0.23	79.79	80.31	0.66
13	850	0.75	48000	0.84	0.8321	0.94	92.11	93	0.96	92.15	92.79	0.70
14	850	0.75	22000	0.88	0.8896	1.09	95.18	94.5	0.72	90.92	90.07	0.94
15	850	0.5	35000	0.67	0.6713	0.19	90.7	91.42	0.80	88.86	92.38	3.96
				**MAPE**		**0.78**			**1.04**			**1.59**

**Table 7:** Comparison of theoretical model predictions and experimental findings.

**Objective Function:** **Max (H_2_/CO** **and Max (H_2_ yield)** **and Max (CO yield)**		**Variables**					
		**T**	${{CH}_{4}:CO}_{2}$	**SV**	**H_2_/CO**	**H_2_ Yield %**	**CO Yield%**
	Criteria	value	value	value	Max.	Max.	Max.
	Theoretical	850	0.932	22,000.009	1	91.505	89.376
	Experimental	850	0.932	22,000	1	91.92	89.16

**Table 8:** Comparison of catalytic activity of other catalysts reported in the previous literature.

Catalyst	Catalyst Wt (g)	GHSV (L/h.g_cat_)	RT (°C)	TOS (h)	$Y_{H_{2}}$ (%)	Ref.
10Ni1Y/Al	0.05	120	550	5	35	[54]
10Ni1RhY/Al	0.05	120	650	5	45	[54]
Ni0.75Sr/Al_2_O_3_	0.6	3.6	700	6	72	[55]
5Ni-SP-OA	0.1	24	700	6	45	[56]
7.5Ni-SiO_2_-Imp	0.1	24	700	6	32	[56]
NiPd-SiO_2_-Imp	0.1	24	700	6	88	[56]
Ni-20/MSN	0.02	180	700	3	71	[57]
20Ni/Si-MCM-41_RHA	0.02	30	700	4	38	[58]
20Ni/Si-MCM-41_RHA	0.02	30	800	24	50	[58]
5Ni/ZSM-5	0.15	28	700	18	27	[50]
5Ni2Ce/ZSM5	0.15	28	800	5	68	[50]
5Ni3Si/Al	0.1	42	700	~7	62	[59]
5Ni3W/Al	0.1	42	700	~7	62	[59]
5Ni3TiAl_2_O_3_	0.1	42	700	7	30	[59]
5Ni3MoAl_2_O_3_	0.1	42	700	7	39	[59]
10Ni/ZrO_2_	0.05	48	700	0.5	45	[60]
5Ni/ZrO_2_	0.1	42	700	7	43	[61]
5Ni1Ce/ZrO_2_	0.1	42	700	~7	47	[61]
5Ni/15La85Zr	0.1	42	700	7	84	[62]
5Ni/9La91Zr	0.1	42	700	~7	80	[61]
5Ni2.5Ce/9La91Zr	0.1	42	700	~7	87	[61]
5Ni/9La91Zr	0.1	42	700	~7	58	[63]
5Ni1Gd/9La91Zr	0.1	42	700	~7	80	[63]
5Ni1Cr/9La91Zr	0.1	42	700	~7	81	[63]
10Ni/CeO_2_	0.15	40	750	12	12	[64]
5Ni/CeO_2_-ZrO_2_ (Ce/Zr = 60/40)	0.15	40	700	7	57	[65]
5Ni/9W91Zr	0.1	42	700	~7	43	[66]
5Ni2.5Ce/9W91Zr	0.1	42	700	7	78	[66]
5Ni15YZrO_2_	0.1	42	700	7	55	[67]
5Ni/15Y85Zr	0.1	42	700	7	64	[68]
5Ni/13Y77Zr	0.1	42	700	~7	67	[69]
5Ni2Ce/13Y77Zr	0.1	42	700	~7	80	[69]
5Ni4Ba/13Y77Zr	0.1	42	800	~7	80	[70]
5Ni4Ho/13Y77Zr	0.1	42	700	~7	84	[71]
La_0.6_Ce_0.4_Ni_0.9_Zr_0.1_O_3_	0.1	42	800	7	83	[72]
CeNi_0.9_Zr_0.01_Y_0.09_O_3_	0.1	42	800	7	85	[72]
5Ni/FHY	0.1	15	800	30	75	[73]
5Ni/CBV3024E	0.1	42	700	5	44	This work
5Ni/CBV3024E	0.1	22	850	5	92	This work

$Y_{H_{2}}$ = Hydrogen yield, Cat. = Catalyst, wt = Catalyst weight, RT = Reaction temperature, TOS = Time on stream.

## Data Availability

Data are contained within the article.

## Supplementary Materials

The following supporting information can be downloaded at: https://www.mdpi.com/article/10.3390/nano14151320/s1, Figure S1: XRD of (A) fresh and reduced-5Ni/CP810B, (B) fresh and reduced-5Ni/CBV10A, (C) fresh and reduced-5Ni/CBV20A. Figure S2: The pore size distribution of (A) reduced-5Ni/CBV3024E, (B) reduced-5Ni/CP810B25, (C) reduced-5Ni/CBV10A, (D) reduced-5Ni/CBV20A. Figure S3: Raman spectra of (A) fresh and reduced-5Ni/CBV3024E, (B) fresh and reduced-5Ni/CBV810B, (C) fresh and reduced-5Ni/CBV10A, (D) fresh and reduced-5Ni/CBV20A. Figure S4: SEM images of (A) fresh 5Ni/CBV3024E at a magnification of 1000×, (B) fresh 5Ni/CBV3024E at a magnification of 5000×, (C) fresh 5Ni/CBV20A at a magnification of 3000×, (D) fresh 5Ni/CBV20A at a magnification of 6500×, (E) fresh 5Ni/CP810B at a magnification of 4000×, (F) fresh 5Ni/CP810B at a magnification of 6500×. Figure S5: TGA profiles of used samples after 5 h of reaction. Table S1. A comparison of the present work activity with the results mentioned in the previous literature.
