# Supplementary material for: Ni-Based Molecular Sieves Nanomaterials for Dry Methane Reforming: Role of Porous Structure and Active Sites Distribution on Hydrogen Production
Source: Nanomaterials (Basel). 2024 Aug 5;14(15):1320. doi: 10.3390/nano14151320 (PMC11313978; doi:10.3390/nano14151320)
Supplement: Supplementary file 1 [file nanomaterials-14-01320-s001.zip › nanomaterials-3097388-supplementary.pdf]

Ni-Based Molecular Sieves Nanomaterials for Dry Methane Reforming: Role of Porous Structure and Active Sites Distribution on Hydrogen Production

Ahmed S. Al-Fatesh [1], Ahmed A. Ibrahim [1], Anis H. Fakeeha [1], Ahmed I. Osman [2,*], Yousef M. Alanazi [1], Fahad Saleh Almubaddel [1,*] and Ahmed E. Abasaeed [1]

1 Chemical Engineering Department, College of Engineering, King Saud University, P.O. Box 800, Riyadh 11421, Saudi Arabia; aalfaesh@ksu.edu.sa (A.S.A.-F.); aidid@ksu.edu.sa (A.A.I.); anishf@ksu.edu.sa (A.H.F.); yalanazi1@ksu.edu.sa (Y.M.A.); abasaeed@ksu.edu.sa (A.E.A.);

2 School of Chemistry and Chemical Engineering, Queen's University Belfast, Belfast BT9 5AG, Northern Ireland, UK

* Correspondence: Correspondence: aosmanahmed01@qub.ac.uk (A.I.O.); falmubaddel@ksu.edu.sa (F.S.A.)

Supporting information

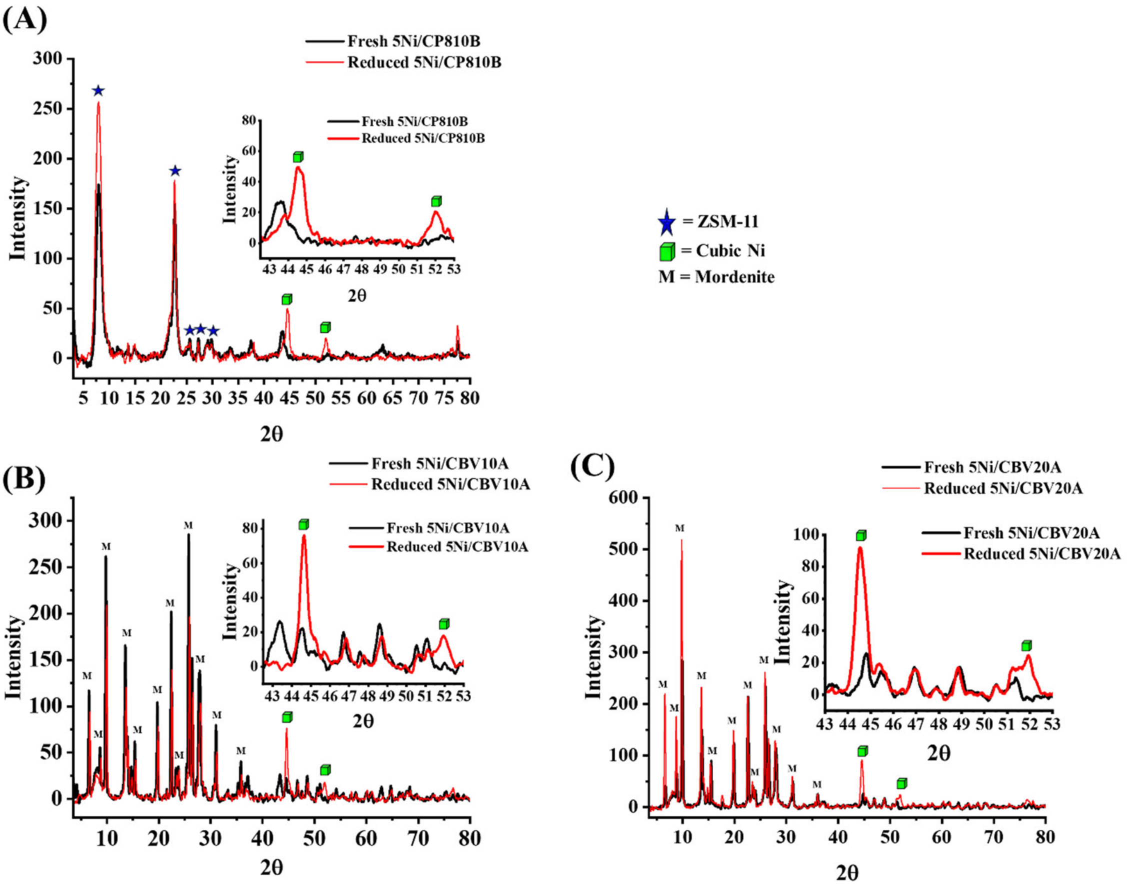

Figure S1. XRD of (**A**) fresh and reduced-5Ni/CP810B (**B**) fresh and reduced-5Ni/CBV10A (**C**) fresh and reduced-5Ni/CBV20A.

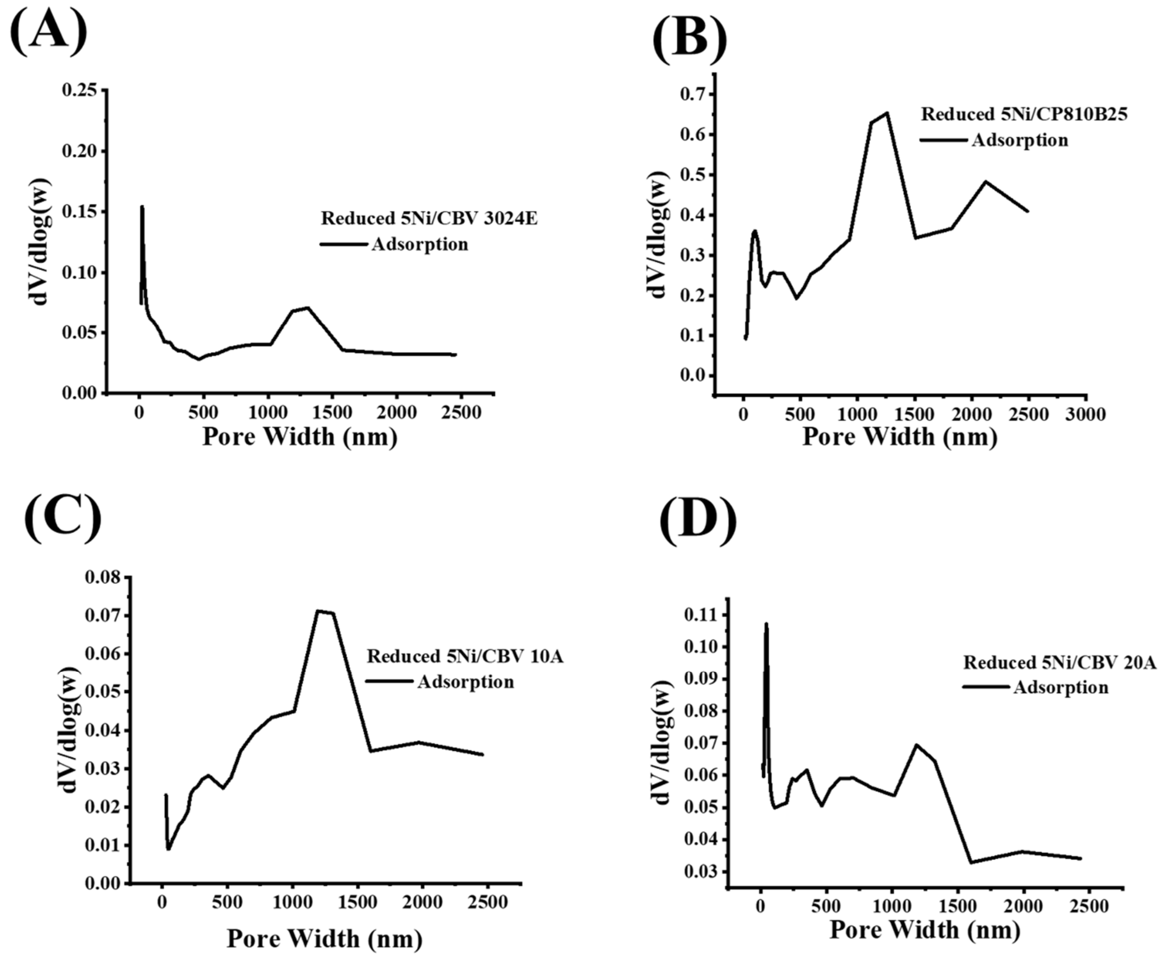

Figure S2. The pore size distribution of (**A**) reduced-5Ni/CBV3024E (**B**) reduced-5Ni/CP810B25 (**C**) reduced-5Ni/CBV10A (**D**) reduced-5Ni/CBV20A.

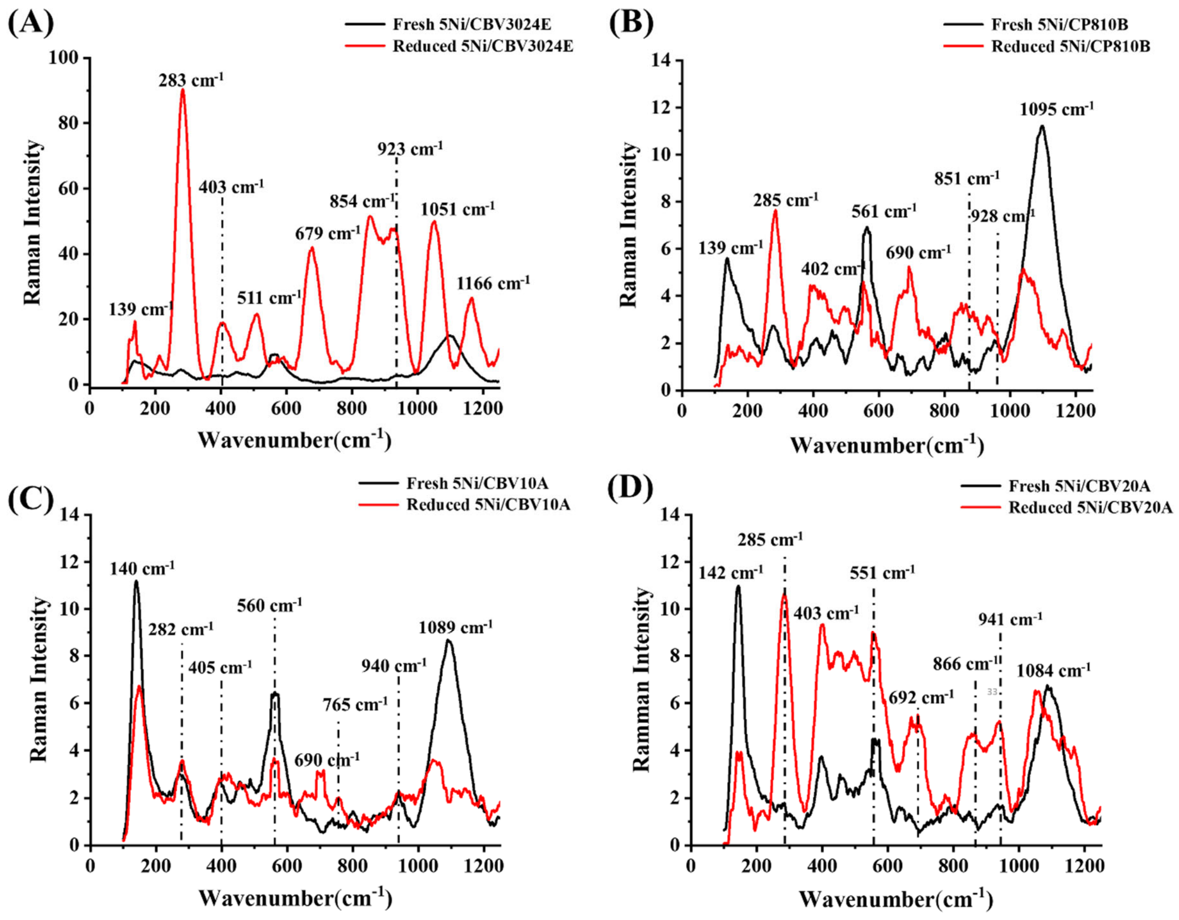

Figure S3. Raman spectra of (**A**) fresh and reduced-5Ni/CBV3024 E (**B**) fresh and reduced-5Ni/CBV810B (**C**) fresh and reduced-5Ni/CBV10A (**D**) fresh and reduced-5Ni/CBV20A.

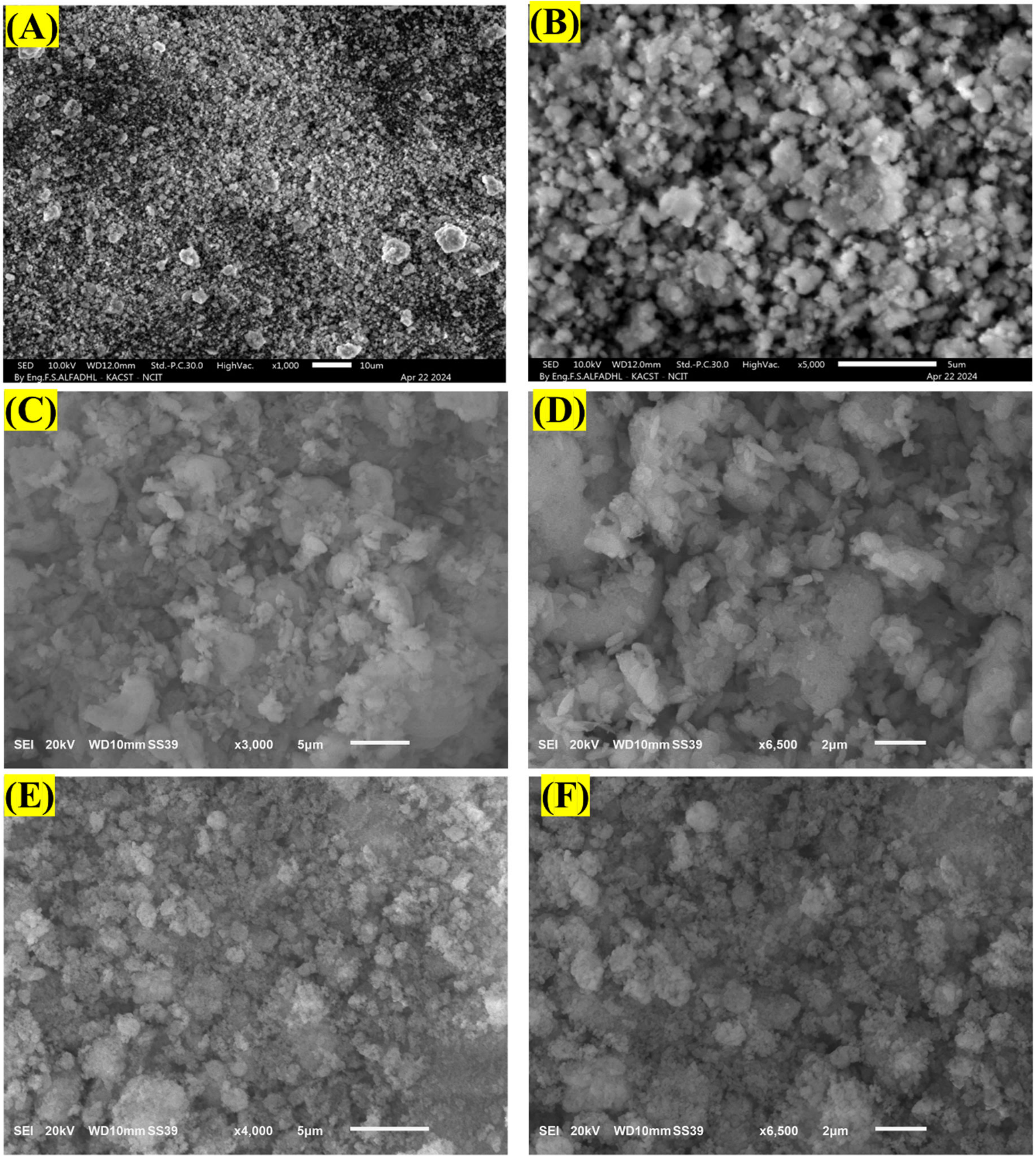

Figure S4. SEM images of (**A**) fresh 5Ni/CBV3024E at a magnification of 1000×. (**B**) Fresh 5Ni/CBV3024E at a magnification of 5000× under high vacuum. (**C**) Fresh 5Ni/CBV20A at a magnification of 3000×. (**D**) Fresh 5Ni/CBV20A at a magnification of 6500×. (**E**) Fresh 5Ni/CP810B at a magnification of 4000×. (**F**) Fresh 5Ni/CP810B at a magnification of 6500×.

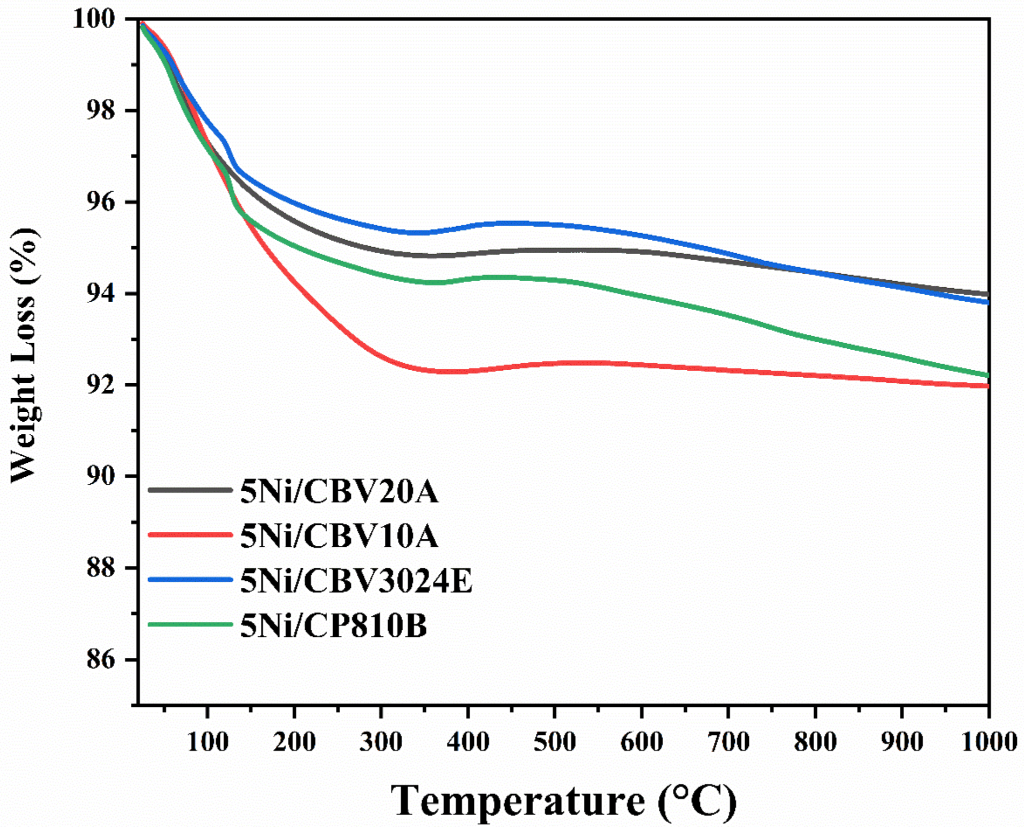

Figure S5. TGA profiles of used samples after 5h of reaction.
